# Risk communication and adaptive behaviour in flood-prone areas of Austria: A Q-methodology study on opinions of affected homeowners

**DOI:** 10.1371/journal.pone.0233551

**Published:** 2020-05-29

**Authors:** Marie-Sophie Attems, Matthias Schlögl, Thomas Thaler, Magdalena Rauter, Sven Fuchs

**Affiliations:** Department of Civil Engineering and Natural Hazards, Institute of Mountain Risk Engineering, University of Natural Resources and Life Sciences, Vienna, Austria; Fukushima Medical University School of Medicine, JAPAN

## Abstract

Adaptive behaviour has become a crucial aspect in current flood risk management strategies across the globe, especially in response to potential consequences of flood hazards and facing challenges of climate change. There are several factors which influence the motivation to implement flood risk management strategies such as property-level flood risk adaptation (PLFRA) measures. This paper assesses and evaluates the role of risk communication, which is a vital and overarching driver or barrier in the successful implementation of PLFRA measures. We explored this issue through a bootstrapped Q-methodology with 20 residents in the urban area of Graz, Austria, who have been affected by flood events in the past. Additionally, semi-structured interviews concerning risk communication were conducted with the participants to understand the preferred risk communication modes. The results show that respondents have a high level of perceived self-efficacy (most have implemented PLFRA measures), that there is general distrust in public protection measures and that there is a high understanding of residual risk. Considering the communication modes preferred by a majority of respondents, face-to-face interaction with unbiased experts is more attractive than online applications. Additionally, citizens want to be engaged in decision-making processes concerning public protection measures in their area. This calls for participatory processes in flood risk management which involve mutual knowledge transfer and social learning.

## 1. Introduction

Due to the effects of climate change and increased exposure of assets in flood-prone areas, losses due to flood hazards are continuously increasing in many regions around the globe [1–3]. In this context it is widely discussed that structural flood alleviation measures, such as dykes and retention basins implemented by public agencies, may not always be sufficient to reduce associated risks to an acceptable level [4–6]. Thus, resulting residual risk (technical or human-induced failure of structural alleviation measures) and the question of how to deal with it, is increasing on the political agenda. Apart from the on-going discussion on increased public budgets for flood alleviation it has repeatedly been argued that private households shall engage in private protection in order to decrease losses by future flood hazard events and increase their resilience to flood impacts [5, 7–9]. Many scholars emphasised that non-structural measures such as property-level flood risk adaptation (PLFRA) measures should be implemented to complement structural flood alleviation measures in flood risk management [10–12]. Globally, there are several examples of how to implement PLFRA measures, ranging from flood avoidance (elevation of buildings or amphibious structures), wet flood-proofing (allowing flood waters to penetrate buildings and minimising associated damage), dry flood-proofing (sealing building openings from water inlet) to barrier systems (temporary and permanent), some of them also in the context of nature-based flood risk management solutions [10, 12–15]. There are several aspects that influence the implementation of PLFRA measures, of which risk communication is considered a vital driver [16–18]. As risk communication should be targeted at specific user groups in order to effectively reach the receivers [19–23], opinions on flood-related topics need to be addressed. Based on past literature, variables which are most strongly associated with adaptive behaviour are the perceived self-efficacy, the outcome efficacy of adaptive actions, negative affect and whether others engage in adaptive actions [5]. Considering that there is a vast amount of literature on adaptive behaviour and influencing variables, there is still a research gap in keeping adaptive behaviour at a high level and continuing the motivation of affected people to stay informed. Hence, the aim of this paper is to identify opinions of stakeholders affected by floods, taking the city Graz, Austria, as an example. The stakeholder opinion groups were formed based on several variables which influence adaptive behaviour by applying Q-methodology. The sample included residents who have experience with floods and have largely been in contact with relevant experts concerning PLFRA measures and retention basins being built in their area. Many have been informed about flood risk management projects through community meetings, the city website, brochures and word-of-mouth recommendation. Thus, communication modes have been mainly conducted in a top-down, one-way direction manner, although there have been personal interactions with selected experts and the mentioned community meetings. Additionally, the paper analyses the communication modes preferred by these specific groups. Based on the assessment, appropriate recommendations for risk communication strategies are suggested, which should be implemented in policy and risk governance arrangements of comparable urban areas. This paper aims at exploring (1) different opinion groups that can be observed in the case study area, (2) communication modes which are preferred by affected residents within different opinion groups and lastly (3) how risk communication can be adapted to specific needs and perceptions of influencing factors.

The paper is organised as follows: Section 2 discusses risk communication and variables which influence adaptive behaviour. Section 3 provides an overview of the case study area, the used method and description of the data analysis. Section 4 presents and discusses the key results of the data analysis and interpretation of the three different factors. Additionally, section 4 gives recommendations for appropriate risk communication strategies based on the results. Finally, section 5 provides a conclusion and future outlook.

## 2. Risk communication and adaptive behaviour

Within international scientific literature, there are several theories and models which are used to identify the factors that positively influence adaptive behaviour of people living in areas at risk, such as the Protection Motivation Theory (PMT) [24, 25], the Person-relative-to-Event theory (PrE) [26], the Protective Action Decision Model (PADM) [27] and the conceptual framework of the Social Amplification of Risk [28], to name the most prominent. Within all these models, risk communication is recognised as a vital driver influencing the response of stakeholders to risk. Under certain conditions, risk communication can influence individual adaptive behaviour [18–21, 29, 30], and therefore, risk communication is considered as an overarching variable with respect to the implementation of PLFRA measures in flood risk management. Considering risk communication, trust in the information source or the expert giving technical advice increases the level of preparedness in many cases [31–33]. The information sources vary from community members, flood action groups [34, 35], neighbours, emergency and relief organisations to the government [36]. Additionally, the media plays a vital role in transmitting and amplifying risks in a community [28]. Therefore, social learning should be enhanced, by including affected stakeholders in decision-making processes [37]. This also involves the use of participatory approaches [37] and mutual knowledge transfer [38]. Trust is a vital building block in flood risk communication as it functions as a mediator to encourage adaptive behaviour [39–41]. Once trust in the communication source is lost, however, it is difficult to regain. Trust can also have negative effects. For example, the reliance on public flood protection sometimes results in a lower willingness to adapt [42]. Hence, the trust in public protection can promote or hinder adaptive behaviour, depending on whether the measures support adaptive behaviour (e.g., warning systems) or reduce the need to adapt on a private level (e.g., retention basins) [5]. In literature, it is argued that the degree of trust in risk managers and flood protection measures is connected to a lack of risk perception of residents at risk [43]. Furthermore, the reliance on government compensation and the perceived responsibility of the government to handle flood risks relates negatively to the willingness of homeowners to adapt on a private level [44].

Several studies depict that there is increasing preparedness in connection with past damage experiences due to flood events [11, 21, 45–48]. Especially where events have self-reference (direct experience), preparedness is larger as people see themselves as possible future victims. As a consequence, residents without flood experience often underestimate the severity of flood events [49]. As this is often the case, there are affected people who do not prepare for floods [50]. A reason for this is that flood experience alone does not always lead to an increase of adaptive behaviour [11]. Risk perception is largely discussed in literature and is in some cases considered to be an important variable influencing peoples’ willingness and ability to adapt and prepare in risk prone areas [19, 51]. Risk perception means the perceived severity and probability of a prevalent or future threat and resulting damages [52], in the case of this analysis a flood event. Several studies assume that low risk awareness is the cause for insufficient preparedness towards disasters [19, 41]. Nevertheless, knowledge about a hazard and thereby high risk perception does not always lead to appropriate preparation [53]. While risk perception correlates with past hazard experiences [54] it will not be sufficient to heighten adaptive behaviour [55]. A review by Bubeck, Botzen [51] states that risk communication should concentrate on explaining the effectiveness of PLFRA measures and how to implement such, as this can keep the motivation to adapt at a high level. Proper information on PLFRA measures and residual risk is especially relevant for new and therefore non-experienced members of a community at risk, as these often have lower levels of risk perception [46]. Thus, knowledge capacities are also vital and can influence adaptive behaviour. This includes knowledge about hazard and risk as well as the understanding of how to prepare for a potential hazard by implementing PLFRA measures. This also includes the understanding of probabilities, flood maps and knowledge on actors concerned with natural hazards [22, 37, 38]. By creating or fostering knowledge capacities, the individual understanding of how to act during a flood event and to understand information communicated can be heightened [56]. However, some studies found that knowledge about hazards does not necessarily correlate with protective behaviour [51].

The social environment can additionally influence adaptive behaviour. This includes the capacity to connect with neighbours and to build up relationships which are helpful in case of flood events. Social networks which are very strong can influence the individual perception of risk. Members of such networks might share similar information on certain prevalent topics and in turn also similar behaviour [55]. Therefore, neighbours can influence adaptive behaviour [56, 57].

Several studies analysed that a higher level of self-efficacy is connected to a higher level of adaptive behaviour [21, 58]. Self-efficacy is the perceived ability to manage specific tasks, in this case to prepare for flood events. This can be different from the actual capability to adapt, which would be the adaptive capacity [5]. Although, all the mentioned variables might be present in residents facing risks of floods, some can still have a feeling of helplessness. This relates to whether or not individuals view hazard events as uncontrollable and if their actions to protect themselves are useful or not. Finally, this leads to the effect of people failing to prepare for future flood events [7, 39, 42, 49].

Considering this literature review on risk behaviour, the most important variables influencing adaptive behaviour towards flood risks were selected. These are used for the interpretation of results gained by the analysis. The variables are categorised as follows: A—Flood experience and risk perception; B—Knowledge capacities; C—Trust in information source and experts; D—Trust in flood protection by the government; E—Social environment, F—Self-efficacy; G—Feeling of helplessness.

## 3. Case study and method

### 3.1. Case study

In order to study both adaptive behaviour and existing communication on flood risks, the city Graz and the surrounding district Graz-Umgebung in Austria was chosen as a case study area. Graz is the second-largest city of Austria encompassing almost 290,000 inhabitants [59]. At EU level the communication of risks is considered to be a responsibility of official bodies as seen in official documents of e.g., the Floods Directive [60]. Flood risk communication in Austria is largely conducted by local governments [56, 61] and several key actors are involved in flood risk management including the federal government, the nine provinces, and roughly 2100 municipalities [62, 63]. Furthermore, administrative bodies carry out activities that are related to flood risk management namely, (1) the Austrian Service for Torrent and Avalanche Control, (2) the Federal Water Engineering Administration and the (3) Austrian Ministry for Transport, Innovation and Technology (at federal state level and provincial level) [63]. Due to risk communication practices, these authorities are partly responsible for a feeling of high safety among citizens who perceive to be at a lower risk [36].

**Fig 1:**
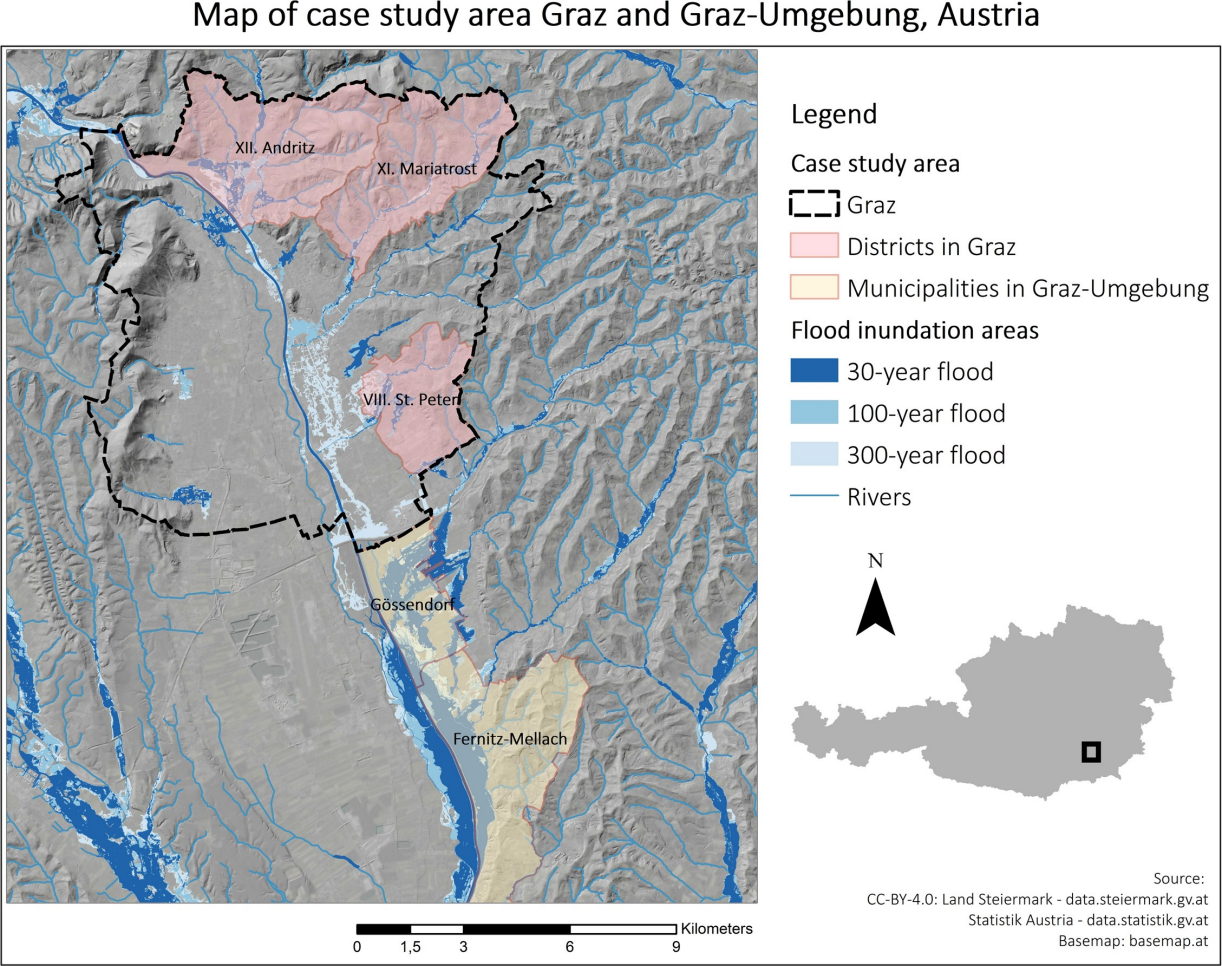
Map of case study area Graz and Graz-Umgebung in Austria, including flood inundation areas.

Graz includes 52 streams and several channels and ditches with a total length of 270 km of which about 125 km are located within the urban area of the city [64]. In the case study area, several single-family homes and smaller apartment blocks are exposed and numerous properties are located directly along tributaries which are feeding the receiving stream (Mur river). Settlements have constantly spread along the streams of Graz and urbanisation processes are still continuing as also seen in Fuchs, Keiler et al. [65]. Graz has experienced several floods in the last decades (1975, 1989, 1996 and 2005) [64] and damages by flood events increased steadily [66]. Damages caused by the 2005 flood event added up to approximately 5 million € and the districts which were affected most strongly were Andritz and St. Peter [64]. As in 2005, typically one rainfall cell centres over Graz and surroundings, resulting in an increased discharge and an overtopping of the majority of streams originating from smaller tributaries in the urban area [67]. The main reason for flood events in these smaller tributaries are thunderstorms in the summer months as well as heavy rainfall on already saturated soil [68]. The main challenges concerning flood risks in Graz and Graz-Umgebung are limited cross sections of streams, leading to insufficient flood discharge capacities. Therefore, the “Special Programme–The Streams of Graz” was implemented in the years 2009–2015, aiming at an improvement of flood protection in the city Graz [64]. The construction of retention basins has subsequently been initiated and in some cases already finalised. Due to past flood events and the construction of retention basins, the risk perception, opinions on risk management and communication is expected to be diverse among affected residents. Fig 1 shows the case study areas within Graz and Graz-Umgebung in Austria, including flood inundation areas.

### 3.2. Q-methodology

For the construction of opinion groups among the affected residents in Graz, Q-methodology was applied. Q-methodology, originally introduced by Stephenson [69], is a method used to study human subjectivity, as it bears the possibility to interview individuals in an interactive way. This method does not have the goal to measure the spread of views in a population, but rather to identify shared viewpoints in order to explain variety [70]. The goal is not to find representatives of a population, but rather to focus on respondents, who are well-informed on a topic and acquire different viewpoints [71]. It is both a quantitative and qualitative technique and thereby solves several qualitative research dilemmas, as it gives a certain structure to the process of analysing qualitative data [72]. Compared to interviewing, Q-methodology is largely reproducible to group different perspectives and it enables the combination of quantitative and qualitative data. In many cases it is applied in combination with interviews, as it gives room for flexibility and creativity of the researcher by integrating the research subject in an interactive way [70]. A standard factor analysis, collectively known as R-methodology, analyses the correlations between variables (e.g., height, age, etc.) and subjects (e.g., respondents). Thus, in R-methodologies, response patterns are analysed and thereby reveal if the valuation of one variable is connected to the valuation of another variable in the same subject. Q-methodology, however, analyses the correlations between respondents, who are defined as variables and it is therefore an inverted factor analysis [73, 74].

Research on adaptive behaviour in flood risk management has been largely conducted using face-to-face surveys, telephone surveys or similar interviewing processes [e.g., 42, 49, 58, 75]. Q-methodology is an exploratory technique [74] and has been widely used in diverging fields of research, such as nuclear risk management [76], environmental studies and policy research [71, 77–80], conservation science [80], ecosystem services [81], agriculture [82], renewable energy [83, 84], health economics [85] and health communication [86]. Only few studies, however, have been using Q-methodology in the area of flood risk management [87–89]. Q-methodology is based on using so-called Q-samples (statements) and P-sets (respondents) to create different factors. These elements will be discussed in the following sections.

#### 3.2.1. Q-samples: Statements

The Q-samples are a collection of items or statements which create a thought and reaction. There are two distinctions of Q-samples: (1) naturalistic Q-samples and (2) ready-made Q-samples [90]. Based on literature review (see section 2), using publications in the field of risk communication and risk behaviour, as well as newspaper articles with opinions on flood risk management, categories which influence adaptive behaviour were established [70]. Therefore, a so-called concourse (sum of viewpoints and perspectives of the research topic) was generated using ready-made materials based on literature rather than following the naturalistic approach where statements would have been generated through interviews beforehand [90]. More than 60 statements on relevant topics of flood risk management were created. After reanalysing the statements through pre-tests with people who are unfamiliar with Q-methodology, 51 statements were chosen as main statements to be used for this study. Omitted statements were either not understandable or included to some extent repeated information found in other statements. The statements are part of seven different variables (A-G) concerned with risk communication and protective behaviour (see section 2). All statements were constructed in German, as this was the main language of respondents and were later translated for this publication (original statements can be seen in S1 Table).

#### 3.2.2. P-set: Respondents

The P-set is defined as the respondents in the Q-methodology [91]. The P-set was chosen based on the flood experience of respondents, resulting in a certain amount of overlap in opinions. A large number of respondents has been in contact with representatives of the city Graz due to the construction of retention basins in their area. All of the respondents directly or indirectly experienced floods, however, with varying extent of damage to their property. Hence, knowledge and perception of risk were expected to be high. Compared to survey techniques, Q-methodology does not require a large population sample in order to reach stable statistical results [73, 90, 92]. Considering insights by Webler and Daniels [73], it is recommended to have more statements than respondents aiming at a 1:3 ratio, which means that there should be one participant for every three statements. Consequently, for this analysis 51 statements resulted in a minimum of 17 required respondents [73]. In order to decrease biases and extend opinions, a final number of 20 respondents were included in the P-set. Fourteen respondents were sampled through the help of a representative of the city Graz, who contacted affected homeowners personally. The remaining six respondents were gained through snowball sampling by homeowners [73]. Table 1 summarises the characteristics of the respondents. The majority of respondents were male property owners, in the age group of 50–59, 70–79 and 60–69.

**Table 1:** Characteristics of respondents (n = 20).

	Number	Percentage (%)
**Age Group (in years)**		
18–29	0	0
30–39	1	5
40–49	1	5
50–59	7	35
60–69	5	25
70–79	6	30
80+	0	0
**Gender**		
Male	13	65
Female	7	35
**Status of property ownership**		
Owner	19	95
Tenant	1	5

Because the University of Natural Resources and Life Sciences, Vienna, was lacking an ethics committee until June 2019, the respondents gave written and verbal consent by signing a privacy statement which was created by the Legal Department of the university and is based on the General Data Protection Regulation of Austria.

#### 3.2.3. Procedure: Q-sorting and semi-structured interviews

**Fig 2:**
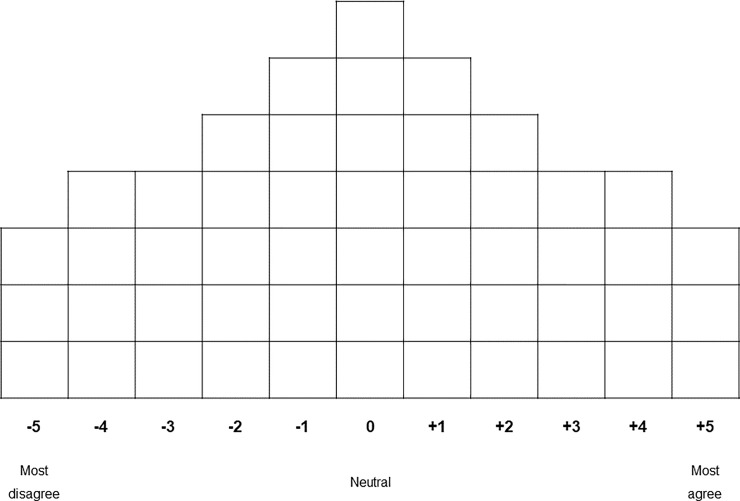
Response grid with 51 boxes given to the respondents for placing the 51 statement cards.

The Q-sorting process was conducted personally by the respondents. After introducing the topic of investigation and the background of the research to each respondent, they received 51 statement cards. Firstly, their task was to read and understand the statements and thereafter sort these into three piles (agree, disagree, neutral) [91]. During this task, respondents could ask questions about the statements in order to clarify any misunderstandings that may have emerged. Respondents were audio-recorded which helped to ensure representative results for the data analysis. After completion, individuals were asked to sort the statements in the given response grid, with one box for each of the 51 statement cards (see Fig 2).

For the Q-sorting process a forced distribution was chosen on a scale from -5 to +5 (most disagree to most agree) as seen in Fig 2 [81]. The response grid was shaped as a quasi-normal distribution, in which lesser number of statements are found in the extremes [93, 94]. In Q-methodology, there is no statistical difference within the vertical sorting. Once the sorting was completed the choices of sorting were openly discussed with the respondents. The final result was the distribution of statements made by a respondent, the so-called Q-sort. Additionally, semi-structured interviews were conducted including four questions, especially focusing on the preferred modes of communication of each participant regarding flood hazards and possible adaptation measures. The questions included: (1) Have you implemented adaptation measures? If yes, which ones? (2) How have you been informed about adaptation measures and flood risks? (3) Were these forms of communication appropriate for your needs? If yes, why? If not, why not? (4) How would you like to be informed in the future? The recorded discussions and the consequent semi-structured interviews were transcribed and coded using the programme “f4transkript” and “f4analyse”. The coding was based on the statements and on the questions asked. Furthermore, inspecting the different measures taken by the respondents on their property was also an important input within the analysis.

#### 3.2.4. Data analysis

To improve the internal validity and robustness of this study, the standard data analysis of Q-methodology was extended by including a bootstrap as proposed by Zabala and Pascual [93]. The data analysis consists of several steps (see a detailed explanation in S1 File), in which the bootstrap already applies at an early stage. The bootstrap was originally introduced by Efron [95] and has since been applied within different statistical approaches in multiple disciplines [96–101], including Q-methodology studies [102–104]. A bootstrap imitates the sampling process from the given population by resampling the original data with replacement for a given amount of times [98]. The resamples can be seen as alternative estimates for the original sample. In this case, the Q-sorts were replaced as a whole [93]. In order to reach satisfactory results and to estimate confidence intervals (CIs) and considering the trade-off between reliability and computation time, 5,000 repetitions were performed for this analysis [93]. All data analysis steps were conducted using the R-package ‘qmethod’ [105] in R [106] (see html file in S2 File).

The collected data of the 20 respondents was distributed in a matrix (see S2 Table), which was used to conduct a principal components analysis (PCA), commonly applied in Q-methodology. This revealed the intercorrelation between each Q-sort (respondent). Thereafter, unrotated factors were extracted and organised according to their explained variability [105]. The number of factors to be extracted was decided on the following: (1) the total amount of variability explained, (2) eigenvalues (EV) higher than 1 and (3) at least two Q-sorts per factor which load significantly upon it [91]. To improve the interpretability of the output, a so-called ‘simple structure’ was targeted by rotating the factors using varimax rotation. The result was a matrix of factor loadings [93]. The next step of the analysis included automated ‘flagging’ of the most representative Q-sorts that defined each factor. So-called z-scores were used to specify how much a factor corresponds to a statement. The last step included the identification of distinguished factors and consensus factors, which was based on the z-scores of each statement [93]. Distinguishing statements are statements which rank in a position that significantly differs from the rank in other factors. The opposite are consensus statements, which can in many cases reveal common perspectives on topics, are ambiguous, or expose topics which respondents do not want to give an opinion on [93]. According to Zabala and Pascual [93], stable statements which should be considered for interpretation are (1) statements with a small standard error (SE) and which do not change position in the factors, (2) distinguishing factors which stay distinguishing and (3) Q-sorts which are not ambiguous and are consistent for a given factor. The statistics which were vital for the factor interpretation were the z-scores and the SE of each statement and factor. These results were enhanced by considering the factor scores and by ranking these using a crib sheet as seen by Watts and Stenner [91] (see S3 Table). The transcripts of the discussions and semi-structured interviews of each respondent were additionally used for the interpretation of the factors. The following sections will explain these results in more detail. The factor interpretations use the following notations (as seen in S1 Table): statement ID; factor score; variable (A-G).

## 4. Results and discussion

**Fig 3:**
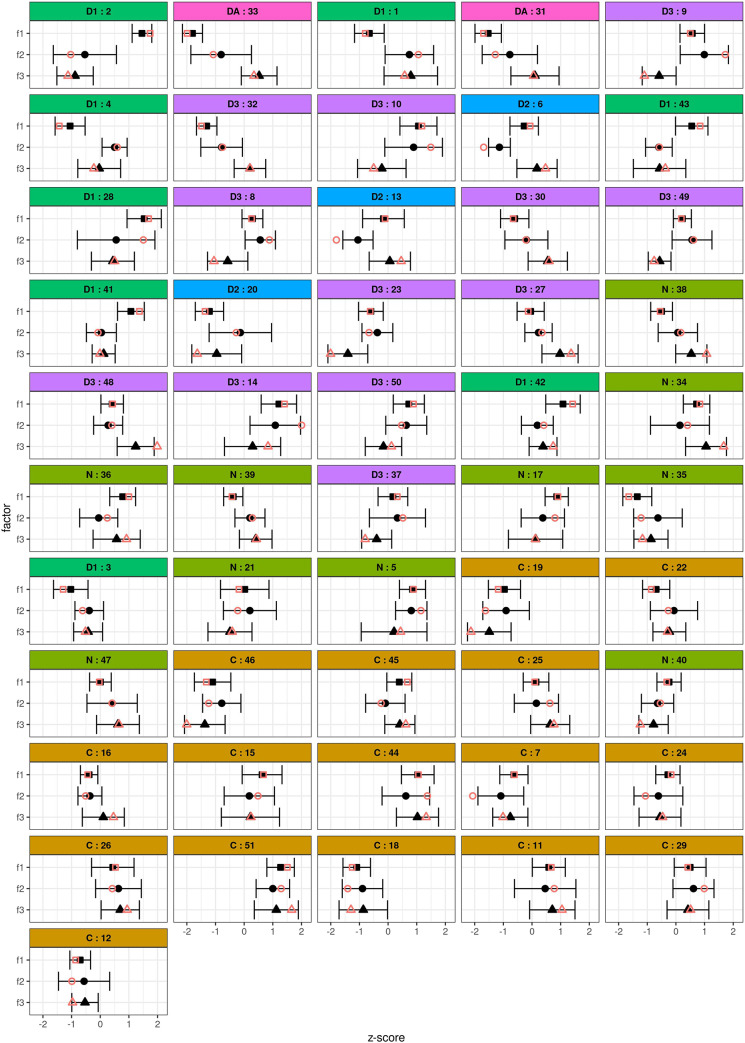
**Statements are ordered based on their z-scores from most distinguished (top) to most consensus (bottom).** The bootstrap z-scores are the means of 5,000 iterations. Error bars show the SE seen in the bootstrap. Filled symbols: bootstrap, hollow symbols: standard. C—Consensus (ochre): None of the differences are significant as all factors give similar scores; D1—Distinguishes factor 1 only (green): the differences between factor 1 and all other factors is significant; D2—Distinguishes factor 2 only (blue): the differences between factor 2 and all other factors is significant; D3—Distinguishes factor 3 only (violet): the differences between factor 1 and all other factors is significant; DA—Distinguishes all (pink): The differences between all pairs of factors are significant; N—Empty string (olive): Statements which do not fulfil any of the other conditions.

The data analysis resulted in three diverging factors of opinion groups, which possessed EVs above 1 (S4 Table) and at least two Q-sorts loading significantly on them. The most important results from the data analysis were the factor scores which display the ‘ideal’ Q-sort for each factor (S1 Table), the factor loadings and flagging frequency (S5 Table), the distinction between consensus and distinguished statements (see Fig 3) and whether the statements were stable within a factor (S6 Table).

### 4.1. Factor scores

The analysis resulted in three factors, which each contain different viewpoints of respondents. Fig 3 displays the statements and their z-scores of the standard Q-analysis as well as the bootstrap variant and the SE for the statements. Fig 3 demonstrates that statements D1 (green) distinguish factor 1 only and consequently the difference between factor 1 and all other factors is significant. Statements D2 (blue) distinguish factor 2 only. Statements D3 (violet) distinguish factor 3 only. Statements DA (pink) distinguish all factors significantly. Statements C (ochre) are consensus statements, where all factors give similar scores to statements. Statements N (olive) are statements which fulfil none of the conditions above. Thereafter, a comparison between standard Q-methodology results and the bootstrap variant has to be made in order to see whether there are unstable statements, which change position to another factor. This can be seen in S6 Table. The results show that statements 4, 7, 8, 9, 13, 14, 16, 17, 19, 21, 22, 24, 25, 28, 29, 31, 32, 34, 35, 36, 38, 39, 40, 45, 46 and 50 change positions to another factor or are insignificant and are therefore unstable statements. These were not used for the final interpretation of factors.

### 4.2. Key factors in risk communication and adaptive behaviour

The selection of statements, which were used for the interpretation of the factors, was based on the results seen in S1 Table, Fig 3, S3 Table and in S6 Table. Thus, the interpretation of the three factors was based on selected statements and their factor scores. In order to comprehensively interpret each factor, the qualitative data was taken into account. To compare results with relevant literature, the variables (A-G) discussed in section 2 were also considered for the interpretation of factors.

There is generally consensus on the experience with temporary barriers, such as sandbags, etc. (Statement 11). Additionally, there is consensus on the idea that one can implement protective measures on one’s own home (Statement 12). In general, there is a rather weak agreement that flood events could be decreased if there were less areas paved (Statement 15). There is also general agreement that there are more flood events than there have been in the past (Statement 18). Although there is a general agreement that damages by floods have risen (Statement 26), the agreement is not very strong. But there is strong consensus about neighbours having had damages by floods (Statement 44), which shows that there is an understanding of the flood consequences in the neighbourhood. Lastly, there is very strong agreement on the idea that citizens should be involved in the decision-making process concerning public flood protection measures in their area (Statement 51). Further statements, which were not consensus statements, were considered for the interpretation of each factor. These show differences in several perspectives. Three factors were explanatory for the gathered data: factor 1 –perceived self-efficacy and distrust in public protection, factor 2 –trust in public protection and satisfied with existing risk communication and lastly factor 3 –low perceived self-efficacy and trust in public protection. A short overview of each factor is given in Table 2 and each factor will be described in greater detail in the following sections.

**Table 2:** Comparison of the main results within each factor.

Factor 1: Perceived self-efficacy and distrust in public protection	Factor 2: Trust in public protection and satisfied with existing risk communication	Factor 3: Low perceived self-efficacy and trust in public protection
• High understanding of residual risk and distrust in public protection measures • Consider hazard zone maps unusable (some grew up in the area) • Communication with neighbours and high trust in them • Do not see functioning communication between public and flood authorities • Do not feel well informed • Want to be actively included in decision-making processes	• High understanding of flood probabilities • Trust in existing public protection measures • Understanding of residual risk but no fear of being affected by potential flood events (contradicting) • Social interaction with neighbours • Content with existing risk communication • Want to be actively included in decision-making processes	• Do not feel prepared for future flood events • Negative consequences of a flood event are considered to be manmade due to urbanisation processes • Trust in public protection • Understanding of residual risk is low • Do not talk to neighbours about floods • Content with existing risk communication • Interested in one-way communication modes (e.g., brochures)

#### 4.2.1. Factor 1: Perceived self-efficacy and distrust in public protection

Factor 1 accounts for 24,62% of explained variance and is defined by seven Q-sorts. For this factor, 13 statements are descriptive. Factor 1 is a group of very self-confident citizens, which are highly informed about floods (Statement 2: 5; B, Statement 3: -3; B). Due to this high knowledge capacity, there is also an understanding of residual risk and distrust in public protection measures (Statement 33: -5; D). Knowing that a potential flood event can cause large damages, they have implemented measures, such as waterproof basement windows (Statement 10: 4; B). They have also used temporary measures, such as sandbags distributed by the fire department (Statement 11: 2; B). This group considers hazard zone maps to be unusable, as the maps are either considered to be incorrect or there is a lack of knowledge on where to find such (Statement 30: -2; C). This might also indicate a lack of knowledge on how to read and use the maps. However, most of the respondents grew up with floods and therefore never needed or wanted to find information on prevalent floods in their area (Statement 1: -2; B). There is distrust in the media concerning the communication of hazards, as this group has witnessed more events than which have been reported (Statement 18: -3; B). This group communicates with their neighbours and talks about past and possible future flood events (Statement 41: 4, Statement 44: 3; E). They trust their neighbours to provide help during a flood, as most have experienced help in the past (Statement 42: 4; E). Although there seems to be a robust communication network within the community, this group does not feel that there is functioning communication between citizens and flood authorities (Statement 43: 2; E). This group neither feels well informed about potential risks nor about the construction processes of retention reservoirs and dykes in their area. The majority does not feel that they were adequately involved in decision-making processes in the past and would want to be actively included in the future (Statement 51: 5; F). Discussions with relevant participants of this group revealed a shared opinion, that young residents are not well informed about prevalent risks when moving to this area, which can lead to new buildings that are not adapted to floods. Thus, according to this group it is vital to raise awareness to flood hazards in the current and new population. Information will have to be communicated more strongly than it currently is, as brochures about PLFRA measures are discarded by people. The most productive way to achieve this is by having community meetings. However, as past community meetings have not been productive, these will have to include unbiased experts which can give technical advice. Considering direct emergency warning, this group was very fond of the text message delivered by the fire department in the past, which communicated upcoming flood warnings. However, this service was discontinued, which made several citizens of this group rather discontent.

#### 4.2.2. Factor 2: Trust in public protection and satisfied with existing risk communication

Factor 2 accounts for 14,95% explained variance and is defined by six Q-sorts and 10 statements. This factor includes a group of citizens with a high understanding of flood probabilities (Statement 6: -5; B) and knowledge on where to find information about flood risks (Statement 1: 3; B). The group includes individuals which are confident about their knowledge about the proximity to the next waterbody that can lead to a flood hazard (Statement 5: 4; B) and they are aware that residual risk is always present (Statement 33: -3; D). There is a willingness, although not very strong, to pay for expert advice concerning PLFRA measures (Statement 37: 2; C). This group is also very social, as there is knowledge about the neighbours’ flood experiences (Statement 44: 4; E). However, the trust in neighbours providing help during a flood event is rather low (Statement 42: 1; E), as neighbours can only help to a certain extent. Yet, there is also trust in existing public protection measures, which creates confidence in the assumption that a potential flood event will not affect them in the future (Statement 2: -3; A). This group does not believe that there is a lack of communication between experts and residents (Statement 43: -2; E), but they consider that citizens should be involved in decision-making concerning flood protection in the area (Statement 51: 4; F). This group shares the opinion that there is enough information available. In their view, it is their own responsibility to find information if needed. This group is not very interested in community meetings to discuss floods, as they do not consider this type of information transfer to be useful. Largely, this group implemented PLFRA measures based on the experience of their network or personal knowledge. Using this network and building on previous knowledge, these citizens found relevant information which was necessary for them to implement PLFRA measures. The implementation was thus not triggered by risk communication efforts.

#### 4.2.3. Factor 3: Low perceived self-efficacy and trust in public protection

The last factor accounts for 12,80% explained variance and is defined by five Q-sorts and 13 statements. This factor shows that flood hazards are defined as a serious threat to the person, family and home (Statement 23: -5; A). Citizens in this group do not have the feeling that they can prepare for a possible future flood event (Statement 49: -2; F). The group hence does not consider e.g., watertight cellar windows to be a useful measure (Statement 10: -2; B). They think risks either do not concern them or they are unaware about the benefit of the measure. Nevertheless, negative consequences of flood events are considered to be man-made (Statement 27: 4; B), as new houses are being built in flood prone areas. This group uses hazard zone maps for information (Statement 30: 2; C), however, they have difficulties to understand the probability of a flood occurring (Statement 6: 1; B). Therefore, their understanding of residual risk is rather low (Statement 33: 0; D, Statement 2: -3; A), which is amplified by trust in existing retention reservoirs and dykes. Consequently, this group is not willing to pay for an expert to give technical advice on PLFRA measures (Statement 37: -2; C). This group rather considers guiding material, such as a brochure on possible protection measures to be useful (Statement 48: 5; B), as they have received similar types of information in the past. The communication channel and information on floods do not necessarily have to be face-to-face. This group does not talk to their neighbours about future flood events, a reason being that they may deny floods from occurring (Statement 41: -1; E). Nevertheless, this group feels like they can rely on the help of their neighbours in case of a flood event (Statement 42: 2; E) as this was the case in past. Thus, this group relies on their social network, to provide help during a flood event. Overall, this group does not see that there is a lack of communication between experts and residents (Statement 43: -1; E), although the factor score is not very strong.

### 4.3. Role of risk communication in enabling PLFRA

The results suggest no clear tendency towards specific risk communication modes per se. Considering that the respondents of this analysis are property-owners which have largely implemented PLFRA measures, a certain format of risk communication is needed to keep risk awareness high and thereby continuously motivate adaptive behaviour.

Considering all three factors, the format of risk communication which can keep risk perception at a high level could be designed as a face-to-face interactive process in the shape of community hall meetings, workshops or excursions. To promote certain decisions, the communicators should be independent, unbiased experts, or individuals which residents trust [56], because government agencies were often perceived to be biased in promoting specific protection technologies, as also seen in literature [31]. Low self-efficacy observed in residents partly resulted from the feeling of being entirely protected by retention reservoirs and were also connected to the disregard of information on floods. Thus, trust in public protection is linked to low perceived self-efficacy of residents [43, 107]. Hence, it is vital to foster the understanding of residual risk among residents. Furthermore, trust in experts, media and governmental decisions are vital drivers for hazard mitigation [53]. Results indicate that residents who have been in contact with experts concerning public protection measures in their area were very content with the communication process. Generally, there is trust in the fire brigade using digital communication modes as warning systems as well as neighbourhood networks, which provide help during flood events [36]. Therefore, different risk communication channels should be made use of, rather than applying highly sophisticated technical risk communication systems. This also means making use of participatory decision-making processes, which include residents at an early stage and incorporating local expertise [35]. Results also revealed that there are new building developments in flood prone areas, which attract younger residents who often show low levels of risk perception [46]. Consequently, they do not possess an extended social network in the community, compared to residents who have been living in the area over a longer period of time. Thus, the social network is vital to foster individual preparedness [55]. Including these younger residents of the area in the analysis would be an improvement to understand their preferred modes of communication.

## 5. Conclusions

This paper discussed various viewpoints of residents in flood-prone areas concerning PLFRA measures and risk communication. All respondents have implemented PLFRA measures, ranging from highly technical measures to very simple, homemade solutions. Thus, the willingness to prepare on the private level was present in all examples. Overall, the results demonstrate three major perspectives of homeowners, which create an understanding of the diversity of perceptions among affected people: (1) perceived self-efficacy and distrust in public protection, (2) trust in public protection and content with existing risk communication and (3) low perceived self-efficacy and trust in public protection. Homeowners in factor 1 exhibited a large knowledge capacity and a sound understanding of residual risk, while homeowners in factor 2 placed a large amount of trust in public protection measures even if they understood the basics beyond residual risk. Hence, factor 2 might not be as educated concerning flood risk management as factor 1. In contrast, people connected to factor 3 were quite pessimistic about the idea of preparing for flood events. This factor was found to be highly dependent on trust in public protection and–simultaneously–was also not interested in learning more about flood hazards.

Generally, however, risk awareness was high among respondents and the idea of residual risk has been largely understood. There was general awareness on flood events and the damage of recent flood events. Moreover, the understanding of the threat to the entire community has been understood, as neighbourhood connections were present. It can be concluded that there was a general understanding of risk levels the respondents are living with. Largely, the respondents felt responsible for their own protection, but still wanted to be involved in decision-making processes concerning protection measures in their area. Consequently, it has to be assumed that the respondents were part of a highly educated group within society with respect to flood adaptation.

The findings of this analysis can be integrated in improving communication strategies implemented in other areas prone to floods. The three factors included several topics which are relevant to increase the motivation of homeowners to prepare on a private level. Within a certain risk communication process, risk awareness could be fostered, especially focusing on the understanding of residual risk. In addition, the possibilities on PLFRA measures should be communicated, especially for residents which are new to the community. Moreover, results demonstrate that participatory decision-making processes would be vital to enhance the understanding of risk and PLFRA measures. This process has to take into account specific needs of younger residents which need to be comprehensively informed and properly included in risk communication processes. However, not all affected residents can be accessed using this communication process, as there will always be a group of citizens which is not interested in face-to-face interaction. Consequently, one-way communication in the form of a website, brochures or text messages will still be necessary to reach the majority of residents at risk.

Considering the method used, it became apparent that Q-methodology is an effective approach to interactively collect information on different perspectives. Compared to conducting solely interviews or surveys, the respondents were triggered to think about the statements given to them in a more creative way. Nevertheless, the Q-sorting process and the construction of statements was time consuming. Overall, the P-set was rather homogenous, as the participants were largely residents at the age of 60 and above. Considering the data analysis, it became clear that combining qualitative and quantitative data can be very challenging. This was especially evident for the factor interpretation. Moreover, the choice of statements used for the analysis can influence the resulting factors. This has to be considered when conducting Q-methodology studies. Nevertheless, applying a bootstrap enhanced the reliability of the used data and improved the results of the study.

In sum, the information obtained by this analysis can be included in risk governance practices to improve current risk communication strategies in flood prone areas. This contributes to increase the social capacity of areas at risk. If implemented on a larger scale, comprehensive communication strategies can be created to contribute to more resilient urban areas.

## Supporting information

S1 Table Factor scores for three factors, including statements used for the Q-sorts and the thematic group they are associated with. Variables: A) Flood experience, Risk perception, B) Knowledge capacities, C) Trust in information source and experts, D) Trust in flood protection and government, E) Social environment, F) Self-efficacy, G) Feeling of helplessness.
(PDF)

S2 Table Matrix of responses gained from the Q-sorting processes. This is the collected data from the 20 respondents (P1-20) during the Q-sorts and is distributed in a matrix in which statements are represented as the rows and respondents are represented in the columns. The cells contain the rating of the statements on a scale from -5 to +5. This was the basis for the data analysis.
(PDF)

S3 Table Cribsheet system used to compare the factor scores of each statement in factors 1 to 3. (PDF)

S4 Table Summary of results including the Eigenvalues of each factor. (PDF)

S5 Table Comparison of standard and bootstrapped results for Q-sort factor loadings. F: factors. Bold: ^1^flagged Q-sorts; ^2^SE>0,2; ^3^frequency of flagging in the bootstrap >0,8.
(PDF)

S6 Table Distinguishing and consensus statements for standard Q-methodology and its bootstrapped variant. The table shows whether statements stay distinguished or consensus (TRUE) and thus do not change their factor score. If the position changes to another factor, this statement is marked with FALSE and shaded in the colour grey.
(PDF)

S1 File A detailed description of the steps used for the data analysis. (PDF)

S2 File Application of Q-methodology: Analysing risk communication and adaptive behaviour in flood-prone area. This html displays the R-code used for the analysis and explains relevant steps applied during the analysis.
(HTML)
