# Supplementary material for: Risk communication and adaptive behaviour in flood-prone areas of Austria: A Q-methodology study on opinions of affected homeowners
Source: PLoS One. 2020 May 29;15(5):e0233551. doi: 10.1371/journal.pone.0233551 (PMC7259652; doi:10.1371/journal.pone.0233551)
Supplement: S1 Table — Variables: A) Flood experience, Risk perception, B) Knowledge capacities, C) Trust in information source and experts, D) Trust in flood protection and government, E) Social environment, F) Self-efficacy, G) Feeling of helplessness. (PDF) [file pone.0233551.s001.pdf]

**S1 Table. Factor scores for three factors, including statements used for the Q-sorts and the thematic group they are associated with.** Variables: A) Flood experience, Risk perception, B) Knowledge capacities, C) Trust in information source and experts, D) Trust in flood protection and government, E) Social environment, F) Self-efficacy, G) Feeling of helplessness.

| ID | Variable | Statement (English) | Original statement (German) | Factor Scores | | |
|---|---|---|---|---|---|---|
| | | | | Factor 1 | Factor 2 | Factor 3 |
| **1.** | B | I know where to find information about the flood risk of my property. | Ich weiß, wo ich Informationen über das Hochwasserrisiko meines Grundstückes finden kann. | -2 | 3 | 2 |
| **2.** | A | Another flood could affect me in the near future. | In naher Zukunft könnte mich ein Hochwasser betreffen. | 5 | -3 | -3 |
| **3.** | B | If I don't live in a high risk area (red zone) there is no need to implement measures on my house. | Wenn ich außerhalb der roten Gefahrenzone wohne, muss ich am eigenen Haus keine Schutzmaßnahmen implementieren. | -3 | -2 | -2 |
| **4.** | B | There is enough information available on floods. | Es gibt ausreichend Informationen über Hochwasser. | -4 | 2 | -1 |
| **5.** | B | I know the distance to the next water body, which could be at risk of overflowing. | Ich weiß, wie weit mein Haus vom nächsten Gewässer entfernt ist, welches ein Hochwasserrisiko darstellt. | 2 | 4 | 1 |
| **6.** | B | Flood probabilities in Austria are difficult to understand. | Hochwasserwahrscheinlichkeiten werden in Österreich schwer verständlich dargestellt. | 0 | -5 | 1 |
| **7.** | B | Existing information about floods in this area is very difficult to understand. | Vorhandene Informationen über Hochwasser in diesem Gebiet sind nur schwer zu verstehen | -2 | -5 | -3 |
| **8.** | B | I know where to get protection measures for my house. | Ich weiß, woher ich private Schutzmaßnahmen für mein Haus bekomme. | 0 | 3 | -3 |
| **9.** | B | I know how to implement protection measures on my house. | Ich weiß, wie man Schutzmaßnahmen umsetzt. | 1 | 5 | -3 |
| **10.** | B | Implementing watertight cellar windows is an effective measure to decrease the cost of damages on my home by a flood. | Wasserdichte Kellerfenster sind eine wirksame Methode, um Hochwasserschäden zu verringern. | 4 | 4 | -2 |
| **11.** | B | I have used temporary barriers (sand sacks, stop logs, etc.) in the past to keep flood water entering my home. | Ich habe bereits temporäre Schutzmaßnahmen, sowie Sandsäcke, eingesetzt um mein Haus vor eindringendem Wasser zu schützen. | 2 | 2 | 4 |
| **12.** | G | There are no protective measures which I can implement on my house. | Ich kann an meinem Haus keine Schutzmaßnahmen umsetzen. | -2 | -2 | -2 |
| **13.** | B | Private protection measures are too expensive. | Private Schutzmaßnahmen sind zu teuer. | 0 | -5 | 1 |
| **14.** | B | I know which materials were used to build my house | Ich weiß, aus welchen Baumaterialien mein Haus errichtet wurde. | 4 | 5 | 3 |
| **15.** | B | I think that damages by floods could be decreased if there were less areas paved. | Wenn weniger Flächen im Ort betoniert wären, dann könnten Schäden durch Hochwasser an meinem Haus verringert werden. | 2 | 1 | 0 |
| **16.** | B | If the costs of different mitigation measures against floods would be more transparent, I would be more willing to implement measures. | Wenn ich die Kosten möglicher privater Schutzmaßnahmen an meinem Haus besser abschätzen könnte, dann würde ich sie eher umsetzen. | -1 | -1 | 1 |

| ID | Variable | Statement (English) | Original statement (German) | Factor Scores | | |
|---|---|---|---|---|---|---|
| | | | | Factor 1 | Factor 2 | Factor 3 |
| **17.** | B | If protective measures would be subsidized, more people would implement these. | Wenn Schutzmaßnahmen an privaten Grundstücken subventioniert wären, würden mehr Personen Schutzmaßnahmen implementieren. | 3 | 3 | 0 |
| **18.** | C | There are not more floods, there is just more reporting in the media. | Es gibt nicht vermehrt Hochwasserereignisse in den letzten 10 Jahren, aber die Medien berichten häufiger darüber. | -3 | -4 | -4 |
| **19.** | C | Flooding is not as big of a problem, as the media makes of it. | Hochwasser ist ein weniger schweres Problem, als es die Medien darstellen. | -3 | -4 | -5 |
| **20.** | A | The view and access to a waterbody is more important to me than a protective wall. | Mir ist die Aussicht auf und der Zugang zum Wasser so wichtig, dass ich dafür auf eine Schutzmauer verzichte. | -4 | -1 | -4 |
| **21.** | G | The effects of flood are uncontrollable. | Die Auswirkungen eines Hochwassers sind unkontrollierbar | -1 | -1 | -1 |
| **22.** | G | Natural hazards are beyond my personal abilities to act. | Naturgefahren liegen jenseits meiner persönlichen Fähigkeiten zu handeln. | -3 | -1 | -1 |
| **23.** | A | I have more important problems than floods. | Ich habe größere Probleme, als mich mit Hochwasser auseinanderzusetzen. | -2 | -2 | -5 |
| **24.** | B | I am interested in learning more about the floods. | Ich würde gerne mehr über die Entstehung von Hochwasser lernen. | -1 | -3 | -1 |
| **25.** | B | I am worried about the effects of climate change in my community. | Ich mache mir Sorgen um die Auswirkungen des Klimawandels auf diese Gemeinde. | 0 | 2 | 3 |
| **26.** | B | I believe the damages caused by floods have risen in my community over the last decades. | Ich glaube, dass Schäden durch Hochwasser in meiner Gemeinde im letzten Jahrzehnt gestiegen sind. | 1 | 1 | 3 |
| **27.** | B | Flood catastrophes are man-made problems. | Hochwasserkatastrophen sind durch den Menschen verursacht. | 0 | 0 | 4 |
| **28.** | A | I have experienced damages on my home caused by floods. | Ich war bereits von Schäden durch Hochwasser an meinem Haus betroffen. | 5 | 5 | 1 |
| **29.** | C | Weather forecasts are very useful for determining upcoming flood events. | Wettervorhersagen sind eine brauchbare Warnung vor möglichen Hochwassersituationen. | 1 | 3 | 1 |
| **30.** | C | I use the hazard zone maps provided by the government to evaluate my flood risk | Ich verwende die Risikokarten der Gemeinde, um mein persönliches Hochwasserrisiko festzustellen. | -2 | 0 | 2 |
| **31.** | A | There will most probably not occur a flood in the next 10 years in this area. | In den nächsten 10 Jahren wird mit hoher Wahrscheinlichkeit kein Hochwasser in meiner Gemeinde eintreten. | -5 | -4 | 0 |
| **32.** | D | Current safety measures implemented by the government in this town provide sufficient protection. | Schutzmaßnahmen, welche durch die Regierung implementiert wurden bieten ausreichend Schutz. | -4 | -2 | 0 |
| **33.** | D | Thanks to existing protection measures, there is no residual risk in this area. | Dank bereits bestehender Schutzmaßnahmen gibt es in dieser Gemeinde kein verbleibendes Risiko. | -5 | -3 | 0 |
| **34.** | D | The government should take care of the flood risk. | Die Regierung sollte sich um Hochwasserprobleme kümmern. | 2 | 0 | 5 |

| ID | Variable | Statement (English) | Original statement (German) | Factor Scores | | |
|---|---|---|---|---|---|---|
| | | | | Factor 1 | Factor 2 | Factor 3 |
| **35.** | D | In case of a flood, I can rely on the support by the government. | Im Falle eines Hochwassers kann ich mich auf die Regierung verlassen. | -5 | -3 | -4 |
| **36.** | D | Politicians should be more involved with the needs of people concerning floods. | PolitikerInnen sollten sich vermehrt mit den Wünschen der EinwohnerInnen bezüglich Hochwasserproblemen befassen. | 3 | 0 | 3 |
| **37.** | C | I am willing to pay for expert advice on flood protection measures on my home. | Ich bin bereit für die Beratung eines/einer Experten/Expertin bezüglich Hochwasserschutzmaßnahmen Geld zu zahlen. | 1 | 2 | -2 |
| **38.** | C | Personal information given by an expert about flood risks is reliable. | Persönliche Beratung eines/einer Experten/Expertin über Hochwasser ist zuverlässig. | -1 | 0 | 4 |
| **39.** | B | I want to get information about floods through a website. | Ich möchte Informationen über Hochwasser durch eine Webseite erfahren. | -1 | 0 | 0 |
| **40.** | E | I have been in contact with people responsible, concerning protection measures. | Ich stehe bereits in Kontakt mit Personen bezüglich private Schutzmaßnahmen für mein Grundstück. | -1 | -1 | -4 |
| **41.** | E | I talk to my neighbours about their flood experience. | Ich spreche mit meinen NachbarInnen über deren Hochwassererfahrungen. | 4 | 0 | -1 |
| **42.** | E | I can rely on the help of my neighbours in case of a flood event. | Ich kann mich auf die Hilfe meiner NachbarInnen im Falle eines Hochwasserereignisses verlassen. | 4 | 1 | 2 |
| **43.** | E | I think there is a lack of communication between experts and residents. | Ich finde, es herrscht eine fehlende Kommunikation zwischen HochwasserexpertInnen und Einwohnern. | 2 | -2 | -1 |
| **44.** | E | My neighbours have had damages by floods. | Meine NachbarInnen haben bereits Schäden von Hochwasserereignissen erfahren. | 3 | 4 | 4 |
| **45.** | E | My neighbours have implemented protective measures. | Meine NachbarInnen haben private Schutzmaßnahmen implementiert. | 1 | -1 | 2 |
| **46.** | E | I don’t see the need to implement measures at my house, as my neighbours aren’t either. | Ich sehe nicht die Notwendigkeit Schutzmaßnahmen an meinem Haus zu implementieren, da es meine NachbarInnen auch nicht tun. | -4 | -4 | -5 |
| **47.** | B | I want to know if my property is at risk of floods. | Ich möchte wissen ob mein Eigentum durch Hochwasser gefährdet ist. | 0 | 1 | 2 |
| **48.** | B | A guide on possible protective measures would be helpful. | Ein Leitfaden, der über mögliche Schutzmaßnahmen informiert, wäre hilfreich. | 1 | 1 | 5 |
| **49.** | F | I can properly prepare my house for a possible flood event. | Ich kann mein Haus für ein potentielles Hochwasserereignis ausstatten. | 0 | 2 | -2 |
| **50.** | F | I am responsible for my own protection against flood hazards. | Ich bin für meinen eigenen Schutz vor Hochwassergefahren verantwortlich. | 3 | 1 | 0 |
| **51.** | F | I think citizens should be involved in the decision-making concerning flood protection in the area. | EinwohnerInnen sollten an den Entscheidungen bezüglich Schutzmaßnahmen in der Gemeinde teilhaben. | 5 | 4 | 5 |
