# Supplementary material for: Risk communication and adaptive behaviour in flood-prone areas of Austria: A Q-methodology study on opinions of affected homeowners
Source: PLoS One. 2020 May 29;15(5):e0233551. doi: 10.1371/journal.pone.0233551 (PMC7259652; doi:10.1371/journal.pone.0233551)
Supplement: S2 Table — This is the collected data from the 20 respondents (P1-20) during the Q-sorts and is distributed in a matrix in which statements are represented as the rows and respondents are represented in the columns. The cells contain the rating of the statements on a scale from -5 to +5. This was the basis for the data analysis. (PDF) [file pone.0233551.s002.pdf]

S3 Table. Matrix of responses gained from the Q-sorting processes. This is the collected data from the 20 respondents (P1-20) during the Q-sorts and is distributed in a matrix in which statements are represented as the rows and respondents are represented in the columns. The cells contain the rating of the statements on a scale from -5 to +5. This was the basis for the data analysis.

Statement	P1	P2	P3	P4	P5	P6	P7	P8	P9	P10	P11	P12	P13	P14	P15	P16	P17	P18	P19	P20
1	0	4	-2	2	0	-5	-3	4	2	-2	5	0	5	4	3	-3	0	-4	-1	-3
2	-2	-4	4	-5	4	1	-2	-5	-1	3	-2	-4	1	2	4	2	-4	5	5	3
3	-1	-2	-1	-2	-1	0	0	-2	1	-5	-3	-2	1	-2	-5	0	-2	0	-4	-5
4	3	2	-5	0	2	-2	-2	1	0	-3	4	-2	-1	2	0	-3	-2	-2	-5	-3
5	3	3	0	4	4	3	1	1	4	0	0	0	-5	1	4	3	5	2	2	4
6	-2	-3	-4	-3	-3	-3	4	-4	-3	0	2	2	-2	-4	-1	0	0	0	1	2
7	-5	-3	-4	-3	0	-4	-1	-4	-4	-2	-3	-2	-3	-4	-1	-2	-1	-1	-1	1
8	3	2	0	1	2	-3	-4	2	4	3	-2	0	-4	1	2	-2	1	0	0	1
9	1	3	0	0	4	4	-5	5	3	4	-1	3	0	3	2	1	-2	0	1	0
10	5	-1	4	5	4	5	-5	5	-2	-1	0	1	1	3	-2	4	-1	5	4	3
11	-2	1	0	4	5	3	2	4	4	-2	-1	5	2	-2	4	3	4	1	3	2
12	4	-1	-2	-3	-1	-2	-3	-5	-1	-1	-2	-1	0	-4	-1	-1	-1	-1	-4	-2
13	-4	-2	4	4	0	-5	3	-4	-4	3	0	3	0	-2	-1	1	-3	-5	-1	-1
14	1	3	0	3	1	5	4	5	5	4	1	2	3	4	5	3	-4	5	4	0
15	5	-1	5	2	3	1	-1	2	-2	4	3	4	4	-1	1	-2	-4	-2	1	4
16	1	-2	-1	0	-2	0	4	-1	-2	-1	-3	-1	-1	-2	0	-1	2	-3	-1	1
17	-2	2	1	-1	1	5	0	0	3	2	-4	2	2	4	3	5	4	2	1	2
18	-5	-3	-1	-5	-1	1	-2	-1	-5	-4	-3	1	-5	-4	-3	-3	1	-4	-2	-3
19	-4	-4	-3	-4	1	-3	-4	-3	-2	-1	-5	-2	-5	-3	0	-4	-3	-2	-4	-4
20	-4	0	-5	-4	-2	3	-5	4	-4	-5	0	1	-1	-5	-2	-4	-5	-3	-2	-1
21	0	4	5	4	5	-2	0	-3	2	-3	-5	-1	-1	-1	1	5	-1	-3	-2	-2
22	2	4	1	0	0	-1	0	-3	-3	-5	-4	1	1	0	-3	-2	0	-2	-1	-2
23	-1	0	-1	-2	0	-5	-4	0	-3	-3	-4	1	-4	-1	-4	0	-5	-1	0	-2
24	-3	-4	-1	2	-4	-4	1	-1	-1	0	-1	-4	-3	-1	-3	-1	-4	1	0	0
25	4	1	2	-1	1	0	1	-1	4	0	5	4	2	1	-4	1	0	1	0	1

Statement	P1	P2	P3	P4	P5	P6	P7	P8	P9	P10	P11	P12	P13	P14	P15	P16	P17	P18	P19	P20
26	-1	5	-2	2	3	2	1	0	3	0	1	-4	5	-2	1	-5	1	4	3	4
27	1	-1	1	-1	1	2	3	1	1	1	3	-3	5	0	-4	-4	2	0	0	2
28	4	-4	2	3	5	4	-1	3	5	5	1	5	1	5	5	5	3	3	4	3
29	5	0	3	3	-2	4	-1	0	0	2	4	-4	-1	4	0	2	2	3	-3	2
30	-5	1	0	-2	-4	-4	1	1	2	-2	3	0	4	1	-3	-1	-1	-4	-2	3
31	-3	-5	-4	1	-3	-1	0	1	-5	-4	-4	-1	1	-5	-4	-5	3	-3	-3	-5
32	-4	-5	-4	-1	-4	-1	1	0	1	-2	2	3	-2	-1	-2	-5	1	-4	-3	-4
33	-1	-5	-3	3	-5	-3	-2	2	-5	-4	2	3	0	-5	-5	-4	3	-5	-5	-5
34	0	4	3	0	1	3	5	-2	0	1	4	5	3	3	2	4	2	1	2	1
35	-1	1	-5	-4	-1	0	-1	-5	-3	-3	-2	-1	-4	-3	-2	-3	-2	-5	-4	-3
36	3	0	3	-1	0	1	3	-1	0	2	5	4	-2	1	3	4	2	1	3	0
37	-1	-2	-3	-2	-2	-4	-3	4	3	1	0	-5	-2	2	0	0	0	3	2	0
38	0	3	-3	1	-4	2	5	-2	2	-1	2	0	0	0	0	0	0	0	-1	-4
39	0	1	-1	1	-5	0	0	1	1	-1	-1	-3	4	0	-1	1	1	-2	-2	1
40	0	-2	2	-3	-3	-1	-3	-3	-1	0	-1	4	-2	2	0	0	-3	-1	-3	-1
41	2	2	5	-2	-1	-1	-1	-1	1	2	0	-1	2	-3	3	1	1	4	2	5
42	1	0	2	5	-1	2	2	2	0	5	1	2	0	-1	1	-1	0	4	4	5
43	-3	-3	4	-4	-2	-2	4	0	0	0	-5	1	0	0	1	-2	-3	2	5	0
44	2	-1	1	1	3	1	2	3	5	4	1	2	4	5	5	2	5	2	0	5
45	2	1	0	0	-5	-2	2	-2	-1	1	1	-3	-1	0	4	1	3	3	2	-1
46	-3	-1	-2	-5	-3	0	-4	-4	-1	-4	-2	0	-4	-3	-5	2	-5	-1	-5	-4
47	0	5	-2	0	0	-1	0	-2	0	1	-1	-2	3	5	-2	2	5	1	0	-1
48	1	0	2	5	2	0	5	0	1	1	3	0	2	3	2	3	4	-1	1	0
49	2	0	1	-1	2	2	-2	3	-4	2	0	-5	-3	2	-1	0	-1	0	1	-1
50	-2	5	1	1	3	1	2	2	-2	5	2	-3	-3	1	1	-1	-2	4	3	-2
51	4	2	3	2	2	4	3	3	2	3	4	-5	3	0	2	4	4	2	5	4
