# Supplementary material for: Risk communication and adaptive behaviour in flood-prone areas of Austria: A Q-methodology study on opinions of affected homeowners
Source: PLoS One. 2020 May 29;15(5):e0233551. doi: 10.1371/journal.pone.0233551 (PMC7259652; doi:10.1371/journal.pone.0233551)
Supplement: S3 Table — (PDF) [file pone.0233551.s003.pdf]

S4 Table. Cribsheet system used to compare the factor scores of each statement in factors 1 to 3.

Factor 1

Statement	Highest ranked at +5	Factor 1	Factor 2	Factor 3
2	Another flood could affect me in the near future.	5	-3	-3
28	I have experienced damages on my home caused by floods.	5	5	1
51	Citizens should be involved in the decision-making concerning flood protection in the area.	5	4	5
	**Lowest ranked at -5**			
31	There will most probably not occur a flood in the next 10 years in this community.	-5	-4	0
33	Thanks to existing protection measures, there is no residual risk in this area.	-5	-3	0
35	In case of a flood, I can rely on the support by the government	-5	-3	-4
	**Ranked higher in Factor 1 than in other factors**			
7	Existing information about floods in this area is very difficult to understand.	-2	-5	-3
10	Implementing watertight cellar windows is an effective measure to decrease the cost of damages on my home by a flood.	4	4	-2
15	I think that damages by floods could be decreased if there were less areas paved.	2	1	0
17	If protective measures would be subsidized, more people would implement these.	3	3	0
18	There are not more floods, there is just more reporting in the media.	-3	-4	-4
19	Flooding is not as big of a problem, as the media makes of it.	-3	-4	-5
23	I have more important problems than floods.	-2	-2	-5
24	I am interested in learning more about the floods.	-1	-3	-1
36	Politicians should be more involved with the needs of people concerning floods.	3	0	3
40	I have been in contact with people responsible, concerning protection measures.	-1	-1	-4
41	I talk to my neighbours about their flood experience.	4	0	-1
42	I can rely on the help of my neighbours in case of a flood event.	4	1	2
43	I think there is a lack of communication between experts and residents.	2	-2	-1
46	I don't see the need to implement measures at my house, as my neighbours aren't either.	-4	-4	-5
50	I am responsible for my own protection against flood hazards.	3	1	0
	**Ranked lower in Factor 1 than in other factors**			
1	I know where to find information about the flood risk of my property.	-2	3	2
3	If I don't live in a high risk area (red zone) there is no need to implement measures on my house.	-3	-2	-2
4	There is enough information available on floods.	-4	2	-1
11	I have used temporary barriers (sand sacks, stop logs, etc.) in the past to keep flood water entering my home.	2	2	4
16	If the costs of different mitigation measures against floods would be more transparent, I would be more willing to implement measures.	-1	-1	1
20	The view and access to a waterbody is more important to me than a protective wall.	-4	-1	-4
22	Natural hazards are beyond my personal abilities to act.	-3	-1	-1
25	I am worried about the effects of climate change in my community.	0	2	3
26	I believe the damages caused by floods have risen in my community over the last decade.	1	1	3
27	Flood catastrophes are man-made.	0	0	4
29	Weather forecasts are very useful for determining upcoming flood events.	1	3	1

30	I use the hazard zone maps provided by the government to evaluate my flood risk.	-2	0	2
32	Current safety measures implemented by the government in this community provide sufficient protection.	-4	-2	0
38	Personal information given by an expert about flood risks is reliable.	-1	0	4
39	I want to get information about floods through a website.	-1	0	0
44	My neighbours have had damages by floods.	3	4	4
47	I want to know if my property is at risk of floods.	0	1	2
48	A guide on possible protective measures would be helpful.	1	1	5
	**Neither higher nor lower in Factor 1 than in other factors**			
5	I know the distance to the next water body, which could be at risk of overflowing.	2	4	1
6	Flood probabilities in Austria are difficult to understand.	0	-5	1
8	I know where to get protection measures for my house.	0	3	-3
9	I know how to implement protection measures on my house.	1	5	-3
12	There are no protective measures which I can implement on my house.	-2	-2	-2
13	Private protection measures are too expensive.	0	-5	1
14	I know which materials were used to build my house.	4	5	3
21	The effects of flood are uncontrollable.	-1	-1	-1
34	The government should take care of the flood risk.	2	0	5
37	I am willing to pay for expert advice on flood protection measures on my home.	1	2	-2
45	My neighbours have implemented protective measures.	1	-1	2
49	I can properly prepare my house for a possible flood event.	0	2	-2

Factor 2

Statement	Highest ranked at +5	Factor 2	Factor 1	Factor 3
9	I know how to implement protection measures on my house.	5	1	-3
14	I know which materials were used to build my house.	5	4	3
28	I have experienced damages on my home caused by floods.	5	5	1
	**Lowest ranked at -5**			
6	Flood probabilities in Austria are difficult to understand.	-5	0	1
7	Existing information about floods in this area is very difficult to understand.	-5	-2	-3
13	Private protection measures are too expensive.	-5	0	1
	**Ranked higher in Factor 2 than in other factors**			
1	I know where to find information about the flood risk of my property.	3	-2	2
4	There is enough information available on floods.	2	-4	-1
5	I know the distance to the next water body, which could be at risk of overflowing.	4	2	1
8	I know where to get protection measures for my house.	3	0	-3
10	Implementing watertight cellar windows is an effective measure to decrease the cost of damages on my home by a flood.	4	4	-2
17	If protective measures would be subsidized, more people would implement these.	3	3	0
20	The view and access to a waterbody is more important to me than a protective wall.	-1	-4	-4
22	Natural hazards are beyond my personal abilities to act.	-1	-3	-1
23	I have more important problems than floods.	-2	-2	-5
29	Weather forecasts are very useful for determining upcoming flood events.	3	1	1
35	In case of a flood, I can rely on the support by the government	-3	-5	-4
37	I am willing to pay for expert advice on flood protection measures on my home.	2	1	-2
39	I want to get information about floods through a website.	0	-1	0
40	I have been in contact with people responsible, concerning protection measures.	-1	-1	-4
44	My neighbours have had damages by floods.	4	3	4
49	I can properly prepare my house for a possible flood event.	2	0	-2
	**Ranked lower in Factor 2 than in other factors**			
2	Another flood could affect me in the near future.	-3	5	-3
3	If I don’t live in a high risk area (red zone) there is no need to implement measures on my house.	-2	-3	-2
11	I have used temporary barriers (sand sacks, stop logs, etc.) in the past to keep flood water entering my home.	2	2	4
16	If the costs of different mitigation measures against floods would be more transparent, I would be more willing to implement measures.	-1	-1	1
18	There are not more floods, there is just more reporting in the media.	-4	-3	-4
24	I am interested in learning more about the floods.	-3	-1	-1
26	I believe the damages caused by floods have risen in my community over the last decade.	1	1	3
27	Flood catastrophes are man-made.	0	0	4
34	The government should take care of the flood risk.	0	2	5
36	Politicians should be more involved with the needs of people concerning floods.	0	3	3
42	I can rely on the help of my neighbours in case of a flood event.	1	4	2

43	I think there is a lack of communication between experts and residents.	-2	2	-1
45	My neighbours have implemented protective measures.	-1	1	2
46	I don't see the need to implement measures at my house, as my neighbours aren't either.	-4	-4	-5
48	A guide on possible protective measures would be helpful.	1	1	5
51	Citizens should be involved in the decision-making concerning flood protection in the area.	4	5	5
	**Neither higher nor lower in Factor 2 than in other factors**			
12	There are no protective measures which I can implement on my house.	-2	-2	-2
15	I think that damages by floods could be decreased if there were less areas paved.	1	2	0
19	Flooding is not as big of a problem, as the media makes of it.	-4	-3	-5
21	The effects of flood are uncontrollable.	-1	-1	-1
25	I am worried about the effects of climate change in my community.	2	0	3
30	I use the hazard zone maps provided by the government to evaluate my flood risk.	0	-2	2
31	There will most probably not occur a flood in the next 10 years in this community.	-4	-5	0
32	Current safety measures implemented by the government in this community provide sufficient protection.	-2	-4	0
33	Thanks to existing protection measures, there is no residual risk in this area.	-3	-5	0
38	Personal information given by an expert about flood risks is reliable.	0	-1	4
41	I talk to my neighbours about their flood experience.	0	4	-1
47	I want to know if my property is at risk of floods.	1	0	2
50	I am responsible for my own protection against flood hazards.	1	3	0

Factor 3

Statement	Highest ranked at +5	Factor 3	Factor 1	Factor 2
34	The government should take care of the flood risk.	5	2	0
48	A guide on possible protective measures would be helpful.	5	1	1
51	Citizens should be involved in the decision-making concerning flood protection in the area.	5	5	4
	**Lowest ranked at -5**			
19	Flooding is not as big of a problem, as the media makes of it.	-5	-3	-4
23	I have more important problems than floods.	-5	-2	-2
46	I don't see the need to implement measures at my house, as my neighbours aren't either.	-5	-4	-4
	**Ranked higher in Factor 3 than in other factors**			
3	If I don't live in a high risk area (red zone) there is no need to implement measures on my house.	-2	-3	-2
6	Flood probabilities in Austria are difficult to understand.	1	0	-5
11	I have used temporary barriers (sand sacks, stop logs, etc.) in the past to keep flood water entering my home.	4	2	2
13	Private protection measures are too expensive.	1	0	-5
16	If the costs of different mitigation measures against floods would be more transparent, I would be more willing to implement measures.	1	-1	-1
22	Natural hazards are beyond my personal abilities to act.	-1	-3	-1
24	I am interested in learning more about the floods.	-1	-1	-3
25	I am worried about the effects of climate change in my community.	3	0	2
26	I believe the damages caused by floods have risen in my community over the last decade.	3	1	1
27	Flood catastrophes are man-made.	4	0	0
31	There will most probably not occur a flood in the next 10 years in this community.	0	-5	-4
32	Current safety measures implemented by the government in this community provide sufficient protection.	0	-4	-2
33	Thanks to existing protection measures, there is no residual risk in this area.	0	-5	-3
36	Politicians should be more involved with the needs of people concerning floods.	3	3	0
38	Personal information given by an expert about flood risks is reliable.	4	-1	0
39	I want to get information about floods through a website.	0	-1	0
44	My neighbours have had damages by floods.	4	3	4
45	My neighbours have implemented protective measures.	2	1	-1
47	I want to know if my property is at risk of floods.	2	0	1
	**Ranked lower in Factor 3 than in other factors**			
2	Another flood could affect me in the near future.	-3	5	-3
5	I know the distance to the next water body, which could be at risk of overflowing.	1	2	4
8	I know where to get protection measures for my house.	-3	0	3
9	I know how to implement protection measures on my house.	-3	1	5
10	Implementing watertight cellar windows is an effective measure to decrease the cost of damages on my home by a flood.	-2	4	4
14	I know which materials were used to build my house.	3	4	5
15	I think that damages by floods could be decreased if there were less areas paved.	0	2	1
17	If protective measures would be subsidized, more people would implement these.	0	3	3
18	There are not more floods, there is just more reporting in the media.	-4	-3	-4
20	The view and access to a waterbody is more important to me than a protective wall.	-4	-4	-1

28	I have experienced damages on my home caused by floods.	1	5	5
29	Weather forecasts are very useful for determining upcoming flood events.	1	1	3
37	I am willing to pay for expert advice on flood protection measures on my home.	-2	1	2
40	I have been in contact with people responsible, concerning protection measures.	-4	-1	-1
41	I talk to my neighbours about their flood experience.	-1	4	0
49	I can properly prepare my house for a possible flood event.	-2	0	2
50	I am responsible for my own protection against flood hazards.	0	3	1
	**Neither higher nor lower in Factor 3 than in other factors**			
1	I know where to find information about the flood risk of my property.	2	-2	3
4	There is enough information available on floods.	-1	-4	2
7	Existing information about floods in this area is very difficult to understand.	-3	-2	-5
12	There are no protective measures which I can implement on my house.	-2	-2	-2
21	The effects of flood are uncontrollable.	-1	-1	-1
30	I use the hazard zone maps provided by the government to evaluate my flood risk.	2	-2	0
35	In case of a flood, I can rely on the support by the government	-4	-5	-3
42	I can rely on the help of my neighbours in case of a flood event.	2	4	1
43	I think there is a lack of communication between experts and residents.	-1	2	-2
