# Supplementary material for: Risk communication and adaptive behaviour in flood-prone areas of Austria: A Q-methodology study on opinions of affected homeowners
Source: PLoS One. 2020 May 29;15(5):e0233551. doi: 10.1371/journal.pone.0233551 (PMC7259652; doi:10.1371/journal.pone.0233551)
Supplement: S4 Table — (PDF) [file pone.0233551.s004.pdf]

S5 Table. Summary of results including the Eigenvalues of each factor.

	Factor 1	Factor 2	Factor 3
Average reliability coefficient	0.8	0.8	0.8
Number of loading Q-sorts	7	6	5
Eigenvalues (EV)	4.92	2.99	2.56
Percentage of explained variance	24.62	14.95	12.80
Composite reliability	0.97	0.96	0.95
Standard error (SE) of factor scores	0.19	0.20	0.22
