# Supplementary material for: Risk communication and adaptive behaviour in flood-prone areas of Austria: A Q-methodology study on opinions of affected homeowners
Source: PLoS One. 2020 May 29;15(5):e0233551. doi: 10.1371/journal.pone.0233551 (PMC7259652; doi:10.1371/journal.pone.0233551)
Supplement: S5 Table — F: factors. Bold: 1flagged Q-sorts; 2SE>0,2; 3frequency of flagging in the bootstrap >0,8. (PDF) [file pone.0233551.s005.pdf]

S6 Table. Comparison of standard and bootstrapped results for Q-sort factor loadings. F: factors. Bold: [1]flagged Q-sorts; [2]SE>0,2; [3]frequency of flagging in the bootstrap >0,8.

	Standard factor loading[1]			Bootstrapped factor loadings and SE[2]						Flagging frequency[3]		
**Q-sort**	**F1**	**F2**	**F3**	**F1**		**F2**		**F3**		**F1**	**F2**	**F3**
**P1**	0.32	**0.57**	0.20	0.34	0.18	0.42	**0.28**	0.19	**0.25**	0.28	0.42	0.11
**P2**	0.14	**0.51**	0.20	0.16	0.16	0.50	**0.31**	0.18	0.18	0.12	0.64	0.13
**P3**	**0.73**	0.05	0.13	0.68	0.18	0.03	**0.22**	0.07	0.19	**0.93**	0.03	0.03
**P4**	0.18	0.45	**0.51**	0.21	0.16	0.32	**0.25**	0.45	**0.26**	0.04	0.25	0.51
**P5**	0.51	0.53	-0.16	0.51	0.18	0.37	**0.25**	-0.11	**0.21**	0.62	0.26	0.06
**P6**	0.43	**0.46**	0.11	0.41	0.18	0.35	**0.28**	0.09	**0.24**	0.55	0.31	0.06
**P7**	0.30	-0.36	**0.76**	0.22	0.13	-0.17	**0.20**	0.57	**0.23**	0.05	0.05	**0.85**
**P8**	0.06	**0.72**	0.15	0.14	0.17	0.54	**0.34**	0.17	**0.23**	0.07	0.67	0.19
**P9**	0.39	**0.52**	0.32	0.39	0.19	0.41	**0.27**	0.28	**0.22**	0.33	0.37	0.08
**P10**	**0.76**	0.20	0.19	0.73	0.16	0.15	0.17	0.15	0.16	**0.96**	0.02	0.01
**P11**	0.02	0.31	**0.63**	0.07	0.16	0.20	**0.20**	0.59	**0.26**	0.03	0.14	**0.80**
**P12**	0.04	0.04	0.23	0.03	0.19	-0.13	**0.40**	0.14	0.19	0.04	0.24	0.14
**P13**	0.13	0.28	**0.62**	0.14	0.15	0.19	0.18	0.59	**0.26**	0.03	0.08	**0.83**
**P14**	0.45	**0.57**	0.21	0.45	**0.20**	0.43	**0.26**	0.18	**0.22**	0.45	0.38	0.03
**P15**	**0.68**	0.29	0.18	0.66	0.18	0.22	**0.22**	0.14	0.18	**0.88**	0.07	0.02
**P16**	**0.67**	0.21	0.14	0.61	0.19	0.15	**0.28**	0.07	**0.22**	**0.81**	0.11	0.04
**P17**	0.11	0.18	**0.63**	0.09	0.14	0.15	0.16	0.60	**0.27**	0.03	0.06	**0.88**
**P18**	**0.78**	0.23	-0.02	0.74	0.16	0.21	0.16	0.00	0.16	**0.94**	0.03	0.02
**P19**	**0.84**	0.12	0.16	0.77	0.17	0.14	0.17	0.11	0.17	**0.96**	0.02	0.02
**P20**	**0.71**	0.16	0.25	0.69	0.17	0.13	0.17	0.22	0.18	**0.95**	0.02	0.02
