# Supplementary material for: Risk communication and adaptive behaviour in flood-prone areas of Austria: A Q-methodology study on opinions of affected homeowners
Source: PLoS One. 2020 May 29;15(5):e0233551. doi: 10.1371/journal.pone.0233551 (PMC7259652; doi:10.1371/journal.pone.0233551)
Supplement: S6 Table — The table shows whether statements stay distinguished or consensus (TRUE) and thus do not change their factor score. If the position changes to another factor, this statement is marked with FALSE and shaded in the colour grey. (PDF) [file pone.0233551.s006.pdf]

S7 Table. Distinguishing and consensus statements for standard Q-methodology and its bootstrapped variant. The table shows whether statements stay distinguished or consensus (TRUE) and thus do not change their factor score. If the position changes to another factor, this statement is marked with FALSE and shaded in the colour grey.

Statement	Standard	Bootstrap variant	Difference
1	Distinguishes f1 only	Distinguishes f1 only	TRUE
2	Distinguishes f1 only	Distinguishes f1 only	TRUE
3	Distinguishes f1 only	Distinguishes f1 only	TRUE
4	Distinguishes all	Distinguishes f1 only	FALSE
5			TRUE
6	Distinguishes f2 only	Distinguishes f2 only	TRUE
7	Distinguishes f2 only	Consensus	FALSE
8	Distinguishes all	Distinguishes f3 only	FALSE
9	Distinguishes all	Distinguishes f3 only	FALSE
10	Distinguishes f3 only	Distinguishes f3 only	TRUE
11	Consensus	Consensus	TRUE
12	Consensus	Consensus	TRUE
13	Distinguishes all	Distinguishes f2 only	FALSE
14	Distinguishes all	Distinguishes f3 only	FALSE
15	Consensus	Consensus	TRUE
16	Distinguishes f3 only	Consensus	FALSE
17	Distinguishes f3 only		FALSE
18	Consensus	Consensus	TRUE
19		Consensus	FALSE
20	Distinguishes f2 only	Distinguishes f2 only	TRUE
21	Consensus		FALSE
22	Distinguishes f1 only	Consensus	FALSE
23	Distinguishes f3 only	Distinguishes f3 only	TRUE
24	Distinguishes f2 only	Consensus	FALSE
25		Consensus	FALSE
26	Consensus	Consensus	TRUE
27	Distinguishes f3 only	Distinguishes f3 only	TRUE
28	Distinguishes f3 only	Distinguishes f1 only	FALSE
29		Consensus	FALSE
30	Distinguishes f3 only	Distinguishes f3 only	TRUE
31	Distinguishes f3 only	Distinguishes all	FALSE
32	Distinguishes all	Distinguishes f3 only	FALSE
33	Distinguishes all	Distinguishes all	TRUE
34	Distinguishes f3 only		FALSE
35	Consensus		FALSE
36	Distinguishes f2 only		FALSE
37	Distinguishes f3 only	Distinguishes f3 only	TRUE
38	Distinguishes all		FALSE
39	Distinguishes f1 only		FALSE
40	Distinguishes f3 only		FALSE
41	Distinguishes f1 only	Distinguishes f1 only	TRUE
42	Distinguishes f1 only	Distinguishes f1 only	TRUE
43	Distinguishes f1 only	Distinguishes f1 only	TRUE
44	Consensus	Consensus	TRUE
45	Distinguishes f2 only	Consensus	FALSE
46	Distinguishes f3 only	Consensus	FALSE
47			TRUE
48	Distinguishes f3 only	Distinguishes f3 only	TRUE
49	Distinguishes f3 only	Distinguishes f3 only	TRUE
50		Distinguishes f3 only	FALSE
51	Consensus	Consensus	TRUE
