# Supplementary material for: Risk communication and adaptive behaviour in flood-prone areas of Austria: A Q-methodology study on opinions of affected homeowners
Source: PLoS One. 2020 May 29;15(5):e0233551. doi: 10.1371/journal.pone.0233551 (PMC7259652; doi:10.1371/journal.pone.0233551)
Supplement: S1 File — (PDF) [file pone.0233551.s007.pdf]

S2. A detailed description of the steps used for the data analysis

Step 1: The collected data from the 20 respondents during the Q-sorts were distributed in a matrix in which statements are represented in the rows and respondents are represented in the columns (see S2 Table).

Step 2: Using the data matrix, a by-person factor analysis was conducted to find similarities and differences within viewpoints [1, 2]. A multivariate data reduction was applied based on principal components analysis (PCA). When using PCA, the term component would be more correct, however, the term factor is commonly applied in Q-methodology literature and therefore also in this paper. The first output of this analysis was a correlation matrix revealing the intercorrelation between each Q-sort (respondent).

Step 3: Thereafter, the unrotated factors were extracted using PCA. The factors were organised according to their explained variability [3]. The decision for the number of factors included (1) the total amount of variability explained , (2) eigenvalues (EV) higher than 1 and (3) at least two Q-sorts per factor which load significantly upon it [2].

Step 4: There are high loadings found on the most important factors and small loadings on other factors. As this makes the interpretation of factors challenging, factor rotation was used to discriminate between factors [4]. Thus, in order to improve interpretability of the output, a so-called "simple structure" was targeted by rotating the factors. Varimax rotation was employed to differentiate the original variables by extracted factors. The result was a matrix of factor loadings [5]. In order to correct alignment problems in PCA, a correction of the alignment problem was implemented for each bootstrap step, where a resample is drawn and the factor loadings are calculated [5].

Step 5: The next step of the analysis included automated flagging of the most representative Q-sorts which defined each factor. The Q-sorts which are most representative were flagged automatically and not manually, also due to the amount of resamples. Thereby, the differences between factors were

maximised. So-called z-scores were used to specify the association between statements and factors, meaning how much a factor corresponds to a statement. Consequently, the factor scores were acquired by assembling the statements according to the z-scores and matching these statements to the possible values of the original distribution. The last step included the identification of distinguished factors and consensus factors. This was based on the z-scores of each statement [5]. Distinguishing statements are statements which rank in a position that significantly differs from the rank in other factors. The opposite are consensus statements, which can in many cases reveal common perspectives on topics, are ambiguous, or expose topics which respondents do not want to give an opinion on [5].

Step 6: The last step encompassed the interpretation of the factors. Hereby, a combination of results was used: According to Zabala and Pascual [5], stable statements which should be considered for interpretation are (1) statements with a small standard error (SE) and which do not change position in the factors, (2) distinguishing factors which stay distinguishing and (3) Q-sorts which are not ambiguous and are consistent for a given factor. Thus, the statistics which were vital for the interpretation were the z-scores and the SE of each statement and factor. These results were enhanced by considering the factor scores and ranking these using a crib sheet as seen by Watts and Stenner [6] (see S3 Table). As the discussions of each respondent were recorded as well as the consequent semi-structured interviews, this information was additionally used for the interpretation of the factors.

References

1. Dziopa F, Ahern K. A systematic literature review of the applications of Q-Technique and its methodology. Methodology. 2011;7(2):39-55. doi: 10.1027/1614-2241/a000021.
2. Watts S, Stenner P. Doing Q methodology: theory, method and interpretation. Qualitative Research in Psychology. 2005;2(1):67-91. doi: 10.1191/1478088705qp022oa.
3. Zabala A. qmethod: A package to explore human perspectives using Q Methodology. The R Journal. 2014;6(2):163-73.
4. Field A. Discovering statistics using SPSS. 3 ed. London: SAGE Publications Ltd.; 2009.
5. Zabala A, Pascual U. Bootstrapping Q Methodology to improve the understanding of human perspectives. PLoS One. 2016;11(2):e0148087. doi: 10.1371/journal.pone.0148087.
6. Watts S, Stenner P. Doing Q methodological research: theory, method and interpretation. London: Sage; 2012.
