# Supplementary material for: Risk communication and adaptive behaviour in flood-prone areas of Austria: A Q-methodology study on opinions of affected homeowners
Source: PLoS One. 2020 May 29;15(5):e0233551. doi: 10.1371/journal.pone.0233551 (PMC7259652; doi:10.1371/journal.pone.0233551)
Supplement: S2 File — This html displays the R-code used for the analysis and explains relevant steps applied during the analysis. (HTML) [file pone.0233551.s008.html]

R Supplement to: Attems et al. (2019)


# Application of Q-methodology: Analysing risk communication and adaptive behaviour in flood-prone areas

## Application of Q-methodology: Analysing risk communication and adaptive behaviour in flood-prone areas

- Data preparation and exploration
  - Load packages and data
  - Visualize survey results as boxplot
  - Prepare input data
  - Correlation between Q-sorts
- Q methodology
  - Standard Q-methodology
    - Factor loadings, flagged sorts, and scores
    - Distinguishing and consensus statements
  - Bootstrapping
    - Q-sort factor loadings
    - Statements
- Plotting results
  - Tidy data for plotting
  - Figure: z-score by factor
  - Figure: z-score by statement
- R session information

Supplement to: Attems et al. (2019)

20 April, 2020

# Data preparation and exploration

This first section covers data preparation and provides some descriptive statistics of the input data set.

## Load packages and data

First, required packages are loaded, and the data set with qsorts is imported. Note that packages need to be installed via `install.packages("packagename")`.

```
# load packages
library(tidyverse)
library(forcats)
library(gtable)
library(grid)
library(qmethod)
library(knitr)

# ggplot2 color palette
gg_color_hue <- function(n) {
  hues = seq(15, 375, length = n + 1)
  hcl(h = hues, l = 65, c = 100)[1:n]
}
gg_cols <- gg_color_hue(n = 8)[-5]

# path
pth <- "/home/ms/Git/ian-qmethod/"

# import csv file
qdat <- read_csv2(paste0(pth, "dat/qsorts.csv"))
```

## Visualize survey results as boxplot

Results of the sorting can be visualized as boxplot for each of the 51 statements. This gives a first impression of the overall responses and their variability across all 20 respondents.

```
# wide to long format
tmp <- qdat %>% 
  gather(person, value, -Statement) %>% 
  group_by(Statement)

# color coding for boxplots
colcode <- tmp %>%
  summarize(mean = mean(value),
            q25 = quantile(value, 0.25),
            q75 = quantile(value, 0.75)) %>% 
  mutate(col = ifelse(q25 > 0, gg_cols[1],
                      ifelse(q75 < 0, gg_cols[3],
                             gg_cols[5])))

# plot
p1 <- ggplot(tmp, aes(x = factor(Statement), y=value)) +
  geom_boxplot(fill = colcode$col, alpha = 0.9) + 
  theme_bw() +
  xlab("Statement") +
  scale_y_continuous(name = "Sorting", breaks = seq(-5, 5, 1))
p1
```

Statements whose first quartile is larger than zero are color coded as red, while statements whose third quartile is below zero are colored in green. All other statements, where the interquartile range includes zero, are colored in blue.

## Prepare input data

Input data need to be formatted as data frame with statements as rows and respondents’ results (Q-sorts) as columns.

```
# drop statement id
qdat <- qdat %>% 
  select(-Statement)

# print dataset
qdat
```

```
## # A tibble: 51 x 20
##       P1    P2    P3    P4    P5    P6    P7    P8    P9   P10   P11   P12   P13
##    <dbl> <dbl> <dbl> <dbl> <dbl> <dbl> <dbl> <dbl> <dbl> <dbl> <dbl> <dbl> <dbl>
##  1     0     4    -2     2     0    -5    -3     4     2    -2     5     0     5
##  2    -2    -4     4    -5     4     1    -2    -5    -1     3    -2    -4     1
##  3    -1    -2    -1    -2    -1     0     0    -2     1    -5    -3    -2     1
##  4     3     2    -5     0     2    -2    -2     1     0    -3     4    -2    -1
##  5     3     3     0     4     4     3     1     1     4     0     0     0    -5
##  6    -2    -3    -4    -3    -3    -3     4    -4    -3     0     2     2    -2
##  7    -5    -3    -4    -3     0    -4    -1    -4    -4    -2    -3    -2    -3
##  8     3     2     0     1     2    -3    -4     2     4     3    -2     0    -4
##  9     1     3     0     0     4     4    -5     5     3     4    -1     3     0
## 10     5    -1     4     5     4     5    -5     5    -2    -1     0     1     1
## # … with 41 more rows, and 7 more variables: P14 <dbl>, P15 <dbl>, P16 <dbl>,
## #   P17 <dbl>, P18 <dbl>, P19 <dbl>, P20 <dbl>
```

## Correlation between Q-sorts

Since Q-methodology is based on (dis)similarities between respondents, maximum and minimum correlations between different Q-sorts can insightful.

```
# (Pearson) correlation of data
qdat_cor <- cor(x=qdat, use="everything", method="pearson") %>%
  as_tibble

# max correlation
apply(qdat_cor, 2, function(x) round(sort(x, decreasing = TRUE)[2], 2))
```

```
##   P1   P2   P3   P4   P5   P6   P7   P8   P9  P10  P11  P12  P13  P14  P15  P16 
## 0.46 0.40 0.58 0.47 0.50 0.50 0.38 0.47 0.59 0.66 0.41 0.19 0.40 0.59 0.65 0.58 
##  P17  P18  P19  P20 
## 0.40 0.75 0.75 0.67
```

```
# min correlation
apply(qdat_cor, 2, function(x) round(sort(x, decreasing = FALSE)[1], 2))
```

```
##    P1    P2    P3    P4    P5    P6    P7    P8    P9   P10   P11   P12   P13 
##  0.00 -0.08  0.05  0.14 -0.08  0.14 -0.12 -0.12  0.12  0.05  0.01 -0.18  0.09 
##   P14   P15   P16   P17   P18   P19   P20 
##  0.06  0.14  0.05  0.04 -0.18  0.05  0.04
```

# Q methodology

This section contains two main parts:

1. Standard Q-methodology
2. Bootstrapped Q-methodology

## Standard Q-methodology

First, the standard approach of Q-methodology (based on the full input data set) is applied.

### Factor loadings, flagged sorts, and scores

```
# explore the factor loadings
results <- qmethod(as.data.frame(qdat), nfactors=3, rotation='varimax')
```

```
## Q-method analysis.
## Finished on:             Mon Apr 20 10:32:26 2020
## Original data:           51 statements, 20 Q-sorts
## Forced distribution:     TRUE
## Number of factors:       3
## Rotation:                varimax
## Flagging:                automatic
## Correlation coefficient: pearson
```

```
# print factor loadings and flags:
loa.and.flags(results)
```

```
##     fg1   f1 fg2    f2 fg3    f3
## P1      0.32   *  0.57      0.20
## P2      0.14   *  0.51      0.20
## P3    * 0.73      0.05      0.13
## P4      0.18      0.45   *  0.51
## P5      0.51      0.53     -0.16
## P6      0.43   *  0.46      0.11
## P7      0.30     -0.36   *  0.76
## P8      0.06   *  0.72      0.15
## P9      0.39   *  0.52      0.32
## P10   * 0.76      0.20      0.19
## P11     0.02      0.31   *  0.63
## P12     0.04      0.04      0.23
## P13     0.13      0.28   *  0.62
## P14     0.45   *  0.57      0.21
## P15   * 0.68      0.29      0.18
## P16   * 0.67      0.21      0.14
## P17     0.11      0.18   *  0.63
## P18   * 0.78      0.23     -0.02
## P19   * 0.84      0.12      0.16
## P20   * 0.71      0.16      0.25
```

```
# explore standard results
summary(results)
```

```
## Q-method analysis.
## Finished on:             Mon Apr 20 10:32:26 2020
## Original data:           51 statements, 20 Q-sorts
## Forced distribution:     TRUE
## Number of factors:       3
## Rotation:                varimax
## Flagging:                automatic
## Correlation coefficient: pearson
## 
## Factor scores
##    fsc_f1 fsc_f2 fsc_f3
## 1      -2      3      2
## 2       5     -3     -3
## 3      -3     -2     -2
## 4      -4      2     -1
## 5       2      4      1
## 6       0     -5      1
## 7      -2     -5     -3
## 8       0      3     -3
## 9       1      5     -3
## 10      4      4     -2
## 11      2      2      4
## 12     -2     -2     -2
## 13      0     -5      1
## 14      4      5      3
## 15      2      1      0
## 16     -1     -1      1
## 17      3      3      0
## 18     -3     -4     -4
## 19     -3     -4     -5
## 20     -4     -1     -4
## 21     -1     -1     -1
## 22     -3     -1     -1
## 23     -2     -2     -5
## 24     -1     -3     -1
## 25      0      2      3
## 26      1      1      3
## 27      0      0      4
## 28      5      5      1
## 29      1      3      1
## 30     -2      0      2
## 31     -5     -4      0
## 32     -4     -2      0
## 33     -5     -3      0
## 34      2      0      5
## 35     -5     -3     -4
## 36      3      0      3
## 37      1      2     -2
## 38     -1      0      4
## 39     -1      0      0
## 40     -1     -1     -4
## 41      4      0     -1
## 42      4      1      2
## 43      2     -2     -1
## 44      3      4      4
## 45      1     -1      2
## 46     -4     -4     -5
## 47      0      1      2
## 48      1      1      5
## 49      0      2     -2
## 50      3      1      0
## 51      5      4      5
##                                     f1    f2    f3
## Average reliability coefficient   0.80  0.80  0.80
## Number of loading Q-sorts         7.00  6.00  5.00
## Eigenvalues                       4.92  2.99  2.56
## Percentage of explained variance 24.62 14.95 12.80
## Composite reliability             0.97  0.96  0.95
## Standard error of factor scores   0.19  0.20  0.22
```

### Distinguishing and consensus statements

```
# standard error of differences between factors
sed <- as.data.frame(results$f_char$sd_dif)
round(sed,4)
```

```
##        f1     f2     f3
## f1 0.2626 0.2729 0.2865
## f2 0.2729 0.2828 0.2960
## f3 0.2865 0.2960 0.3086
```

```
# distinguishing and consensus statements
dist <- results$qdc %>% 
  mutate_if(is.numeric, round, digits = 2) %>% 
  mutate(statement = 1:nrow(.)) %>% 
  select(statement, everything())
```

#### Table: Distinguishing and consensus statements

```
# table
kable(dist, caption = "Distinguishing and consensus statements",
      align = rep(c("r","l"),4))
```

Distinguishing and consensus statements

| statement | dist.and.cons | f1\_f2 | sig\_f1\_f2 | f1\_f3 | sig\_f1\_f3 | f2\_f3 | sig\_f2\_f3 |
| --- | --- | --- | --- | --- | --- | --- | --- |
| 1 | Distinguishes f1 only | -1.85 | 6\* | -1.37 | \*\*\* | 0.48 |  |
| 2 | Distinguishes f1 only | 2.77 | 6\* | 2.87 | 6\* | 0.09 |  |
| 3 | Distinguishes f1 only | -0.68 | \* | -0.78 | \*\* | -0.10 |  |
| 4 | Distinguishes all | -2.02 | 6\* | -1.21 | \*\*\* | 0.82 | \*\* |
| 5 |  | -0.26 |  | 0.45 |  | 0.71 | \* |
| 6 | Distinguishes f2 only | 1.62 | 6\* | -0.54 |  | -2.16 | 6\* |
| 7 | Distinguishes f2 only | 1.45 | 6\* | 0.40 |  | -1.06 | \*\*\* |
| 8 | Distinguishes all | -0.62 | \* | 1.31 | \*\*\* | 1.94 | 6\* |
| 9 | Distinguishes all | -1.22 | \*\*\* | 1.61 | 6\* | 2.83 | 6\* |
| 10 | Distinguishes f3 only | -0.31 |  | 1.68 | 6\* | 1.99 | 6\* |
| 11 | Consensus | -0.10 |  | -0.37 |  | -0.27 |  |
| 12 | Consensus | 0.13 |  | 0.11 |  | -0.02 |  |
| 13 | Distinguishes all | 1.70 | 6\* | -0.56 | \* | -2.26 | 6\* |
| 14 | Distinguishes all | -0.60 | \* | 0.58 | \* | 1.17 | \*\*\* |
| 15 | Consensus | 0.19 |  | 0.43 |  | 0.24 |  |
| 16 | Distinguishes f3 only | 0.07 |  | -0.90 | \*\* | -0.97 | \*\* |
| 17 | Distinguishes f3 only | 0.10 |  | 0.79 | \*\* | 0.69 | \* |
| 18 | Consensus | 0.16 |  | 0.04 |  | -0.11 |  |
| 19 |  | 0.44 |  | 0.95 | \*\*\* | 0.51 |  |
| 20 | Distinguishes f2 only | -1.09 | \*\*\* | 0.27 |  | 1.36 | \*\*\* |
| 21 | Consensus | 0.04 |  | 0.24 |  | 0.19 |  |
| 22 | Distinguishes f1 only | -0.59 | \* | -0.56 | \* | 0.03 |  |
| 23 | Distinguishes f3 only | 0.04 |  | 1.38 | \*\*\* | 1.34 | \*\*\* |
| 24 | Distinguishes f2 only | 0.89 | \*\* | 0.30 |  | -0.60 | \* |
| 25 |  | -0.53 |  | -0.67 | \* | -0.14 |  |
| 26 | Consensus | 0.11 |  | -0.41 |  | -0.52 |  |
| 27 | Distinguishes f3 only | -0.48 |  | -1.50 | 6\* | -1.02 | \*\*\* |
| 28 | Distinguishes f3 only | 0.18 |  | 1.19 | \*\*\* | 1.00 | \*\*\* |
| 29 |  | -0.57 | \* | -0.10 |  | 0.48 |  |
| 30 | Distinguishes f3 only | -0.45 |  | -1.26 | \*\*\* | -0.81 | \*\* |
| 31 | Distinguishes f3 only | -0.41 |  | -1.75 | 6\* | -1.33 | \*\*\* |
| 32 | Distinguishes all | -0.73 | \*\* | -1.69 | 6\* | -0.96 | \*\* |
| 33 | Distinguishes all | -0.91 | \*\*\* | -2.33 | 6\* | -1.42 | \*\*\* |
| 34 | Distinguishes f3 only | 0.43 |  | -0.83 | \*\* | -1.26 | \*\*\* |
| 35 | Consensus | -0.43 |  | -0.48 |  | -0.05 |  |
| 36 | Distinguishes f2 only | 0.75 | \*\* | 0.08 |  | -0.67 | \* |
| 37 | Distinguishes f3 only | -0.20 |  | 1.12 | \*\*\* | 1.32 | \*\*\* |
| 38 | Distinguishes all | -0.72 | \*\* | -1.62 | 6\* | -0.90 | \*\* |
| 39 | Distinguishes f1 only | -0.71 | \*\* | -0.85 | \*\* | -0.14 |  |
| 40 | Distinguishes f3 only | 0.22 |  | 0.93 | \*\* | 0.70 | \* |
| 41 | Distinguishes f1 only | 1.45 | 6\* | 1.38 | \*\*\* | -0.07 |  |
| 42 | Distinguishes f1 only | 1.01 | \*\*\* | 0.68 | \* | -0.33 |  |
| 43 | Distinguishes f1 only | 1.42 | 6\* | 1.21 | \*\*\* | -0.21 |  |
| 44 | Consensus | -0.31 |  | -0.27 |  | 0.04 |  |
| 45 | Distinguishes f2 only | 0.91 | \*\*\* | 0.05 |  | -0.86 | \*\* |
| 46 | Distinguishes f3 only | -0.09 |  | 0.68 | \* | 0.77 | \*\* |
| 47 |  | -0.45 |  | -0.69 | \* | -0.24 |  |
| 48 | Distinguishes f3 only | 0.03 |  | -1.55 | 6\* | -1.58 | 6\* |
| 49 | Distinguishes f3 only | -0.42 |  | 0.95 | \*\*\* | 1.37 | \*\*\* |
| 50 |  | 0.41 |  | 0.77 | \*\* | 0.36 |  |
| 51 | Consensus | 0.22 |  | -0.15 |  | -0.37 |  |

## Bootstrapping

Second, bootstrapped Q-methodology (based on sampling with replacement) is applied.

```
# bootstrapping
# boots <- qmboots(as.data.frame(qdat), nfactors=3,
#                  nsteps = 5000, rotation='varimax')

# load pre-computed boots result 
boots <- readRDS(paste0(pth, "dat/boots_5k.rds"))

# table summaries for bootstrapped results
qms <- qmb.summary(boots)
```

### Q-sort factor loadings

```
# Q-sorts - look at defining factors
boot_qsort <- round(qms$qsorts, digits=2)
rn <- tibble(qsort = rownames(boot_qsort))
boot_qsort <- bind_cols(rn, boot_qsort) %>% 
  select(-c(f1.bias:f3.bias))
```

#### Table: Factor loadings

```
# table
kable(boot_qsort,
      caption = "Comparison of standard and bootstrap results for
      Q-sort factor loadings.")
```

Comparison of standard and bootstrap results for Q-sort factor loadings.

| qsort | f1.std | f2.std | f3.std | f1.loa | f1.SE | f2.loa | f2.SE | f3.loa | f3.SE | flag.freq1 | flag.freq2 | flag.freq3 |
| --- | --- | --- | --- | --- | --- | --- | --- | --- | --- | --- | --- | --- |
| P1 | 0.32 | 0.57 | 0.20 | 0.34 | 0.18 | 0.42 | 0.28 | 0.19 | 0.25 | 0.28 | 0.42 | 0.11 |
| P10 | 0.76 | 0.20 | 0.19 | 0.73 | 0.16 | 0.15 | 0.17 | 0.15 | 0.16 | 0.96 | 0.02 | 0.01 |
| P11 | 0.02 | 0.31 | 0.63 | 0.07 | 0.16 | 0.20 | 0.20 | 0.59 | 0.26 | 0.03 | 0.14 | 0.80 |
| P12 | 0.04 | 0.04 | 0.23 | 0.03 | 0.19 | -0.13 | 0.40 | 0.14 | 0.19 | 0.04 | 0.24 | 0.14 |
| P13 | 0.13 | 0.28 | 0.62 | 0.14 | 0.15 | 0.19 | 0.18 | 0.59 | 0.26 | 0.03 | 0.08 | 0.83 |
| P14 | 0.45 | 0.57 | 0.21 | 0.45 | 0.20 | 0.43 | 0.26 | 0.18 | 0.22 | 0.45 | 0.38 | 0.03 |
| P15 | 0.68 | 0.29 | 0.18 | 0.66 | 0.18 | 0.22 | 0.22 | 0.14 | 0.18 | 0.88 | 0.07 | 0.02 |
| P16 | 0.67 | 0.21 | 0.14 | 0.61 | 0.19 | 0.15 | 0.28 | 0.07 | 0.22 | 0.81 | 0.11 | 0.04 |
| P17 | 0.11 | 0.18 | 0.63 | 0.09 | 0.14 | 0.15 | 0.16 | 0.60 | 0.27 | 0.03 | 0.06 | 0.88 |
| P18 | 0.78 | 0.23 | -0.02 | 0.74 | 0.16 | 0.21 | 0.16 | 0.00 | 0.16 | 0.94 | 0.03 | 0.02 |
| P19 | 0.84 | 0.12 | 0.16 | 0.77 | 0.17 | 0.14 | 0.17 | 0.11 | 0.17 | 0.96 | 0.02 | 0.02 |
| P2 | 0.14 | 0.51 | 0.20 | 0.16 | 0.16 | 0.50 | 0.31 | 0.18 | 0.18 | 0.12 | 0.64 | 0.13 |
| P20 | 0.71 | 0.16 | 0.25 | 0.69 | 0.17 | 0.13 | 0.17 | 0.22 | 0.18 | 0.95 | 0.02 | 0.02 |
| P3 | 0.73 | 0.05 | 0.13 | 0.68 | 0.18 | 0.03 | 0.22 | 0.07 | 0.19 | 0.93 | 0.03 | 0.03 |
| P4 | 0.18 | 0.45 | 0.51 | 0.21 | 0.16 | 0.32 | 0.25 | 0.45 | 0.26 | 0.04 | 0.25 | 0.51 |
| P5 | 0.51 | 0.53 | -0.16 | 0.51 | 0.18 | 0.37 | 0.25 | -0.11 | 0.21 | 0.62 | 0.26 | 0.06 |
| P6 | 0.43 | 0.46 | 0.11 | 0.41 | 0.18 | 0.35 | 0.28 | 0.09 | 0.24 | 0.55 | 0.31 | 0.06 |
| P7 | 0.30 | -0.36 | 0.76 | 0.22 | 0.13 | -0.17 | 0.20 | 0.57 | 0.23 | 0.05 | 0.05 | 0.85 |
| P8 | 0.06 | 0.72 | 0.15 | 0.14 | 0.17 | 0.54 | 0.34 | 0.17 | 0.23 | 0.07 | 0.67 | 0.19 |
| P9 | 0.39 | 0.52 | 0.32 | 0.39 | 0.19 | 0.41 | 0.27 | 0.28 | 0.22 | 0.33 | 0.37 | 0.08 |

```
# check flagging frequency (clear definers of the factors in which they are flagged)
ff <- 0.8
boot_qsort %>% filter(flag.freq1 >= ff | flag.freq2 >= ff | flag.freq3 >= ff)
```

```
## # A tibble: 11 x 13
##    qsort f1.std f2.std f3.std f1.loa f1.SE f2.loa f2.SE f3.loa f3.SE flag.freq1
##    <chr>  <dbl>  <dbl>  <dbl>  <dbl> <dbl>  <dbl> <dbl>  <dbl> <dbl>      <dbl>
##  1 P10     0.76  0.2     0.19   0.73  0.16   0.15  0.17  0.15   0.16       0.96
##  2 P11     0.02  0.31    0.63   0.07  0.16   0.2   0.2   0.59   0.26       0.03
##  3 P13     0.13  0.28    0.62   0.14  0.15   0.19  0.18  0.59   0.26       0.03
##  4 P15     0.68  0.290   0.18   0.66  0.18   0.22  0.22  0.14   0.18       0.88
##  5 P16     0.67  0.21    0.14   0.61  0.19   0.15  0.28  0.07   0.22       0.81
##  6 P17     0.11  0.18    0.63   0.09  0.14   0.15  0.16  0.6    0.27       0.03
##  7 P18     0.78  0.23   -0.02   0.74  0.16   0.21  0.16  0      0.16       0.94
##  8 P19     0.84  0.12    0.16   0.77  0.17   0.14  0.17  0.11   0.17       0.96
##  9 P20     0.71  0.16    0.25   0.69  0.17   0.13  0.17  0.22   0.18       0.95
## 10 P3      0.73  0.05    0.13   0.68  0.18   0.03  0.22  0.07   0.19       0.93
## 11 P7      0.3  -0.36    0.76   0.22  0.13  -0.17  0.2   0.570  0.23       0.05
## # … with 2 more variables: flag.freq2 <dbl>, flag.freq3 <dbl>
```

### Statements

Calculate results for distinguishing and consensus statements based on the bootstrap

```
# zsc
boot_zsc <- qms$statement %>%
  select(contains("zsc.bts")) %>% 
  # note: qdc greps for "f[0-9]$" resulting from combn, colnames need to follow this pattern
  setNames(nm = c("zsc_f1", "zsc_f2", "zsc_f3"))

# sed
qdat_df <- qdat %>%
  as.data.frame()

boot_loa <- sapply(X = boots$loa.stats, function(x) x$mean)
flagged <- qflag(nstat = nrow(qdat), loa = boot_loa)
qmzsc <- qzscores(qdat_df, nfactors = 3,
                  flagged = flagged,
                  loa = boot_loa)
boot_sed <- qmzsc$f_char$sd_dif

boot_qdc <- qdc(qdat, nfactors = 3, zsc = boot_zsc, sed = boot_sed)$dist.and.cons

# final result: distinguising and consensus statements
qdc_res <- tibble(statement = 1:nrow(qdat),
                  orig = results$qdc$dist.and.cons,
                  boot = boot_qdc) %>% 
  mutate(diff = orig == boot) 

# final table
kable(qdc_res,
      caption = "Distinguishing and consensus statements for
      standard Q-methodology and its bootstrapped variant")
```

Distinguishing and consensus statements for standard Q-methodology and its bootstrapped variant

| statement | orig | boot | diff |
| --- | --- | --- | --- |
| 1 | Distinguishes f1 only | Distinguishes f1 only | TRUE |
| 2 | Distinguishes f1 only | Distinguishes f1 only | TRUE |
| 3 | Distinguishes f1 only | Distinguishes f1 only | TRUE |
| 4 | Distinguishes all | Distinguishes f1 only | FALSE |
| 5 |  |  | TRUE |
| 6 | Distinguishes f2 only | Distinguishes f2 only | TRUE |
| 7 | Distinguishes f2 only | Consensus | FALSE |
| 8 | Distinguishes all | Distinguishes f3 only | FALSE |
| 9 | Distinguishes all | Distinguishes f3 only | FALSE |
| 10 | Distinguishes f3 only | Distinguishes f3 only | TRUE |
| 11 | Consensus | Consensus | TRUE |
| 12 | Consensus | Consensus | TRUE |
| 13 | Distinguishes all | Distinguishes f2 only | FALSE |
| 14 | Distinguishes all | Distinguishes f3 only | FALSE |
| 15 | Consensus | Consensus | TRUE |
| 16 | Distinguishes f3 only | Consensus | FALSE |
| 17 | Distinguishes f3 only |  | FALSE |
| 18 | Consensus | Consensus | TRUE |
| 19 |  | Consensus | FALSE |
| 20 | Distinguishes f2 only | Distinguishes f2 only | TRUE |
| 21 | Consensus |  | FALSE |
| 22 | Distinguishes f1 only | Consensus | FALSE |
| 23 | Distinguishes f3 only | Distinguishes f3 only | TRUE |
| 24 | Distinguishes f2 only | Consensus | FALSE |
| 25 |  | Consensus | FALSE |
| 26 | Consensus | Consensus | TRUE |
| 27 | Distinguishes f3 only | Distinguishes f3 only | TRUE |
| 28 | Distinguishes f3 only | Distinguishes f1 only | FALSE |
| 29 |  | Consensus | FALSE |
| 30 | Distinguishes f3 only | Distinguishes f3 only | TRUE |
| 31 | Distinguishes f3 only | Distinguishes all | FALSE |
| 32 | Distinguishes all | Distinguishes f3 only | FALSE |
| 33 | Distinguishes all | Distinguishes all | TRUE |
| 34 | Distinguishes f3 only |  | FALSE |
| 35 | Consensus |  | FALSE |
| 36 | Distinguishes f2 only |  | FALSE |
| 37 | Distinguishes f3 only | Distinguishes f3 only | TRUE |
| 38 | Distinguishes all |  | FALSE |
| 39 | Distinguishes f1 only |  | FALSE |
| 40 | Distinguishes f3 only |  | FALSE |
| 41 | Distinguishes f1 only | Distinguishes f1 only | TRUE |
| 42 | Distinguishes f1 only | Distinguishes f1 only | TRUE |
| 43 | Distinguishes f1 only | Distinguishes f1 only | TRUE |
| 44 | Consensus | Consensus | TRUE |
| 45 | Distinguishes f2 only | Consensus | FALSE |
| 46 | Distinguishes f3 only | Consensus | FALSE |
| 47 |  |  | TRUE |
| 48 | Distinguishes f3 only | Distinguishes f3 only | TRUE |
| 49 | Distinguishes f3 only | Distinguishes f3 only | TRUE |
| 50 |  | Distinguishes f3 only | FALSE |
| 51 | Consensus | Consensus | TRUE |

#### Table: Statements

```
# define significance thresholds
nstat <- nrow(qdat)
thold.01 <- 2.58/sqrt(nstat)
thold.05 <- 1.96/sqrt(nstat)

# table: bootstrap vs standard results
kable(as_tibble(qms$statements) %>%
        round(digits = 2) %>% 
        mutate(statement = as.character(1:nrow(.))) %>% 
        select(statement, matches("f[1-3].bias|fsc")),
      caption = "Comparison of bootstrap and standard results for statements")
```

Comparison of bootstrap and standard results for statements

| statement | f1.bias | f2.bias | f3.bias | fsc\_f1 | fsc\_f2 | fsc\_f3 | fsc.bts.1 | fsc.bts.2 | fsc.bts.3 | f1.fsc.bias | f2.fsc.bias | f3.fsc.bias |
| --- | --- | --- | --- | --- | --- | --- | --- | --- | --- | --- | --- | --- |
| 1 | -0.14 | 0.31 | -0.21 | -2 | 3 | 2 | -2 | 4 | 4 | 0 | -1 | -2 |
| 2 | 0.28 | -0.50 | -0.25 | 5 | -3 | -3 | 5 | -2 | -4 | 0 | -1 | 1 |
| 3 | -0.27 | -0.23 | -0.09 | -3 | -2 | -2 | -3 | -2 | -1 | 0 | 0 | -1 |
| 4 | -0.38 | 0.09 | -0.19 | -4 | 2 | -1 | -3 | 2 | -1 | -1 | 0 | 0 |
| 5 | 0.03 | 0.34 | 0.22 | 2 | 4 | 1 | 3 | 4 | 1 | -1 | 0 | 0 |
| 6 | 0.21 | -0.55 | 0.30 | 0 | -5 | 1 | -1 | -5 | 0 | 1 | 0 | 1 |
| 7 | 0.02 | -0.98 | -0.26 | -2 | -5 | -3 | -2 | -5 | -3 | 0 | 0 | 0 |
| 8 | -0.03 | 0.32 | -0.48 | 0 | 3 | -3 | 1 | 2 | -3 | -1 | 1 | 0 |
| 9 | -0.07 | 0.73 | -0.52 | 1 | 5 | -3 | 2 | 5 | -3 | -1 | 0 | 0 |
| 10 | 0.12 | 0.60 | -0.28 | 4 | 4 | -2 | 4 | 4 | -1 | 0 | 0 | -1 |
| 11 | 0.09 | 0.32 | 0.34 | 2 | 2 | 4 | 2 | 2 | 4 | 0 | 0 | 0 |
| 12 | -0.16 | -0.43 | -0.44 | -2 | -2 | -2 | -3 | -2 | -2 | 1 | 0 | 0 |
| 13 | 0.06 | -0.75 | 0.40 | 0 | -5 | 1 | 0 | -5 | 0 | 0 | 0 | 1 |
| 14 | 0.20 | 0.92 | 0.54 | 4 | 5 | 3 | 4 | 5 | 1 | 0 | 0 | 2 |
| 15 | 0.05 | 0.30 | 0.02 | 2 | 1 | 0 | 2 | 0 | 1 | 0 | 1 | -1 |
| 16 | -0.05 | -0.15 | 0.35 | -1 | -1 | 1 | -1 | -1 | 0 | 0 | 0 | 1 |
| 17 | 0.04 | 0.42 | -0.01 | 3 | 3 | 0 | 3 | 1 | 0 | 0 | 2 | 0 |
| 18 | -0.16 | -0.52 | -0.43 | -3 | -4 | -4 | -4 | -4 | -4 | 1 | 0 | 0 |
| 19 | -0.22 | -0.72 | -0.64 | -3 | -4 | -5 | -3 | -4 | -5 | 0 | 0 | 0 |
| 20 | -0.15 | -0.14 | -0.68 | -4 | -1 | -4 | -4 | -1 | -4 | 0 | 0 | 0 |
| 21 | -0.20 | -0.42 | 0.08 | -1 | -1 | -1 | 0 | 1 | -2 | -1 | -2 | 1 |
| 22 | -0.17 | -0.20 | -0.07 | -3 | -1 | -1 | -2 | -1 | -1 | -1 | 0 | 0 |
| 23 | -0.02 | -0.29 | -0.60 | -2 | -2 | -5 | -2 | -1 | -5 | 0 | -1 | 0 |
| 24 | 0.11 | -0.45 | 0.09 | -1 | -3 | -1 | -1 | -2 | -2 | 0 | -1 | 1 |
| 25 | -0.04 | 0.47 | 0.13 | 0 | 2 | 3 | 0 | 0 | 3 | 0 | 2 | 0 |
| 26 | 0.09 | -0.22 | 0.24 | 1 | 1 | 3 | 1 | 4 | 3 | 0 | -3 | 0 |
| 27 | -0.09 | 0.12 | 0.39 | 0 | 0 | 4 | 0 | 1 | 4 | 0 | -1 | 0 |
| 28 | 0.15 | 0.95 | 0.06 | 5 | 5 | 1 | 5 | 2 | 2 | 0 | 3 | -1 |
| 29 | -0.08 | 0.38 | 0.09 | 1 | 3 | 1 | 1 | 3 | 2 | 0 | 0 | -1 |
| 30 | -0.05 | -0.02 | 0.05 | -2 | 0 | 2 | -2 | -1 | 2 | 0 | 1 | 0 |
| 31 | -0.16 | -0.51 | -0.05 | -5 | -4 | 0 | -5 | -3 | 0 | 0 | -1 | 0 |
| 32 | -0.18 | 0.03 | 0.00 | -4 | -2 | 0 | -4 | -4 | 0 | 0 | 2 | 0 |
| 33 | -0.19 | -0.27 | -0.18 | -5 | -3 | 0 | -5 | -4 | 2 | 0 | 1 | -2 |
| 34 | 0.12 | 0.26 | 0.62 | 2 | 0 | 5 | 2 | 0 | 5 | 0 | 0 | 0 |
| 35 | -0.30 | -0.59 | -0.30 | -5 | -3 | -4 | -5 | -3 | -4 | 0 | 0 | 0 |
| 36 | 0.22 | 0.30 | 0.34 | 3 | 0 | 3 | 3 | 0 | 3 | 0 | 0 | 0 |
| 37 | 0.15 | 0.19 | -0.40 | 1 | 2 | -2 | 0 | 1 | -1 | 1 | 1 | -1 |
| 38 | -0.05 | 0.10 | 0.54 | -1 | 0 | 4 | -1 | 0 | 2 | 0 | 0 | 2 |
| 39 | -0.04 | 0.09 | 0.03 | -1 | 0 | 0 | -1 | 1 | 1 | 0 | -1 | -1 |
| 40 | -0.08 | 0.10 | -0.46 | -1 | -1 | -4 | -1 | -3 | -3 | 0 | 2 | -1 |
| 41 | 0.30 | -0.12 | -0.13 | 4 | 0 | -1 | 4 | 0 | 0 | 0 | 0 | -1 |
| 42 | 0.34 | 0.22 | 0.35 | 4 | 1 | 2 | 4 | 0 | 1 | 0 | 1 | 1 |
| 43 | 0.29 | 0.02 | 0.20 | 2 | -2 | -1 | 1 | -2 | -2 | 1 | 0 | 1 |
| 44 | 0.02 | 0.75 | 0.30 | 3 | 4 | 4 | 3 | 3 | 4 | 0 | 1 | 0 |
| 45 | 0.28 | -0.15 | 0.21 | 1 | -1 | 2 | 1 | -1 | 1 | 0 | 0 | 1 |
| 46 | -0.22 | -0.46 | -0.63 | -4 | -4 | -5 | -4 | -3 | -5 | 0 | -1 | 0 |
| 47 | -0.04 | 0.00 | 0.04 | 0 | 1 | 2 | 0 | 2 | 3 | 0 | -1 | -1 |
| 48 | 0.02 | 0.13 | 0.75 | 1 | 1 | 5 | 1 | 1 | 5 | 0 | 0 | 0 |
| 49 | -0.04 | 0.05 | -0.19 | 0 | 2 | -2 | 0 | 3 | -2 | 0 | -1 | 0 |
| 50 | 0.16 | -0.16 | 0.28 | 3 | 1 | 0 | 2 | 3 | -1 | 1 | -2 | 1 |
| 51 | 0.24 | 0.29 | 0.54 | 5 | 4 | 5 | 5 | 5 | 5 | 0 | -1 | 0 |

# Plotting results

## Tidy data for plotting

```
# prepare ggplot solution
zsc <- as_tibble(qms$statements) %>% 
  mutate(statement = as.character(1:nrow(.))) %>% 
  select(statement, matches("zsc.bts|SE|zsc.std"))

sds <- abs(apply(zsc[, c(5, 7, 9)], 1, sd))
zsc <- zsc[order(sds), ]

# desired order
ord <- zsc$statement

# tidying: error lines
zsc_se <- zsc %>% 
  mutate(f1_min = f1.zsc.bts - f1.SE,
         f1_max = f1.zsc.bts + f1.SE,
         f2_min = f2.zsc.bts - f2.SE,
         f2_max = f2.zsc.bts + f2.SE,
         f3_min = f3.zsc.bts - f3.SE,
         f3_max = f3.zsc.bts + f3.SE
  ) %>% 
  select(statement, f1_min:f3_max, f1.zsc.bts, f2.zsc.bts, f3.zsc.bts) %>% 
  rename_all(
    list(
      ~stringr::str_replace_all(., '.zsc', '')
    )
  ) %>% 
  gather(key = "key", value = "value", -"statement") %>% 
  separate(key, c("factor", "type")) %>% 
  mutate(statement = ordered(statement, levels = ord)) %>% 
  spread(type, value) %>% 
  rename(value = bts)

# tidying: points
zsc_point <- zsc %>%
  select(statement, contains("zsc")) %>% 
  rename_all(
    list(
      ~stringr::str_replace_all(., '.zsc', '')
    )
  ) %>% 
  gather(key = "key", value = "value", -"statement") %>% 
  separate(key, c("factor", "type")) %>% 
  mutate(statement = ordered(statement, levels = ord),
         factor = factor(factor),
         type = factor(type)) %>% 
  arrange(statement, factor, type)
```

## Figure: z-score by factor

```
# colors for errorbars and points
c1 <- "black"
c2 <- gg_cols[1] # red

# tibble for annotation
ann_text <- tibble(x = -2.25,
                   y = c(9.5, 35),
                   label = c("Consensus", "Distinction"),
                   factor = factor("f1", levels = c("f1", "f2", "f3"))
)

# plot
p2 <- ggplot() +
  geom_errorbarh(data = zsc_se,
                 aes(y = statement, xmax = max, xmin = min),
                 color = c1) +
  geom_point(data = zsc_point,
             aes(x = value, y = statement,
                 color = type, fill = type,
                 shape = interaction(type, factor)),
             size = 2, stroke = 1.5) +
  geom_hline(yintercept = 18.5, linetype = "dashed", color = "dimgrey") +
  scale_color_manual(values = unname(c(c1, c2)), guide="none") +
  scale_shape_manual(values = c(22, 22, 21, 21, 24, 24), guide="none") +
  scale_fill_manual(values = c(c1, NA), guide="none") +
  theme_bw() +
  facet_wrap(~factor) +
  xlab("z-score") +
  geom_text(data = ann_text, aes(x = x, y = y),
            label = ann_text$label, angle = 90)
p2
```

## Figure: z-score by statement

```
# order consensus statements:
cons_order <- c("Consensus",
                "",
                "Distinguishes f1 only",
                "Distinguishes f2 only",
                "Distinguishes f3 only",
                "Distinguishes all"
)

# reverse factor levels for plotting
zsc_rev <- zsc_point %>% 
  mutate(statement = fct_rev(statement),
         factor = fct_rev(factor))

zsc_se_rev <- zsc_se %>% 
  mutate(statement = fct_rev(statement),
         factor = fct_rev(factor))

# order qdc_res
ord_df <- qdc_res %>%
  mutate(statement = factor(statement, levels = ord)) %>% 
  mutate(orig = factor(orig, levels = cons_order),
         boot = factor(boot, levels = cons_order))

# add statements about dist/cons to zsc points
# (needed to color facet header boxes)
zsc_point <- left_join(ord_df,
                       zsc_point %>%
                         mutate(id = 1:nrow(.)),
                       by = "statement") %>% 
  select(id, statement, factor:value, orig:diff) %>% 
  arrange(id) %>%
  mutate(orig = factor(orig))

# fill color for facet strips
col_vec <- gg_cols[2:7]

# color levels
zsc_point <- zsc_point %>% 
  mutate(boot = forcats::fct_recode(boot,
                             C = "Consensus",
                             N = "",
                             D1 = "Distinguishes f1 only",
                             D2 = "Distinguishes f2 only",
                             D3 = "Distinguishes f3 only",
                             DA = "Distinguishes all")) %>% 
  mutate(col_lvl = boot)
levels(zsc_point$col_lvl) <- col_vec

# map color to statement:
col_stat <- zsc_point %>%
  distinct(boot, col_lvl) %>% 
  mutate_if(is.factor, as.character)

# define facet order for 4 column case (4x13)
lvl_order <- levels(zsc_se_rev$statement) %>%
  as.integer()

# number of desired columns in the output ggplot
n_col <- 4

# get remainder
mod <- length(lvl_order) %% n_col
na_fill <- n_col - mod

# vector to matrix
M <- matrix(data = c(lvl_order, rep(NA, na_fill)),
            ncol = n_col,
            byrow = TRUE)
# flip matrix
N <- M[c(nrow(M):1),]

# define facet order
col_grid_ord <- as.vector(t(N)) %>%
  na.omit() %>% 
  factor(., levels = .)

fills <- zsc_point %>%
  distinct(statement, boot, col_lvl) %>%
  mutate(col_lvl = as.character(col_lvl)) %>% 
  mutate(statement = gdata::reorder.factor(statement,
                                           new.order = col_grid_ord)) %>% 
  arrange(statement) %>% 
  .$col_lvl

# new facet label names
labl <- zsc_point %>% 
  distinct(statement, boot) %>% 
  mutate(nam = paste(boot, ":", statement))
statementlabs <- labl$nam
names(statementlabs) <- labl$statement

# plot
p3 <- ggplot() +
  geom_errorbarh(data = zsc_se_rev,
                 aes(y = factor, xmax = max, xmin = min),
                 color = c1) +
  geom_point(data = zsc_point,
             aes(x = value, y = factor,
                 color = type, fill = type,
                 shape = interaction(type, factor)),
             size = 2, stroke = 1.5) +
  scale_color_manual(values = c(c1, c2), guide="none") +
  scale_shape_manual(values = c(22, 22, 21, 21, 24, 24), guide="none") +
  scale_fill_manual(values = c(c1, NA), guide="none") +
  facet_wrap(~statement, ncol = n_col, labeller = labeller(statement = statementlabs)) +
  theme_bw() +
  theme(strip.text.x = element_text(color = "black", face = "bold")) +
  xlab("z-score")

g3 <- ggplot_gtable(ggplot_build(p3))

strip <- grep('strip-t', g3$layout$name)

idx <- vector()
for (i in strip) {
  j <- grep('rect', g3$grobs[[i]]$grobs[[1]]$childrenOrder)
  check <- length(j)
  idx <- c(idx, check)
}
idx <- as.logical(idx)
strip <- strip[idx]

k <- 1
for (i in strip) {
  g3$grobs[[i]]$grobs[[1]]$children[[1]]$gp$fill <- fills[k]
  k <- k+1
}

# draw
grid.newpage()
grid.draw(g3)
```

# R session information

```
sessioninfo::session_info()
```

```
## ─ Session info ───────────────────────────────────────────────────────────────
##  setting  value                       
##  version  R version 3.6.3 (2020-02-29)
##  os       Manjaro Linux               
##  system   x86_64, linux-gnu           
##  ui       X11                         
##  language (EN)                        
##  collate  en_US.UTF-8                 
##  ctype    en_US.UTF-8                 
##  tz       Europe/Vienna               
##  date     2020-04-20                  
## 
## ─ Packages ───────────────────────────────────────────────────────────────────
##  package     * version   date       lib source        
##  assertthat    0.2.1     2019-03-21 [1] CRAN (R 3.6.1)
##  backports     1.1.6     2020-04-05 [1] CRAN (R 3.6.3)
##  bookdown      0.18      2020-03-05 [1] CRAN (R 3.6.3)
##  broom         0.5.5     2020-02-29 [1] CRAN (R 3.6.3)
##  cellranger    1.1.0     2016-07-27 [1] CRAN (R 3.6.1)
##  cli           2.0.2     2020-02-28 [1] CRAN (R 3.6.3)
##  colorspace    1.4-1     2019-03-18 [1] CRAN (R 3.6.1)
##  crayon        1.3.4     2017-09-16 [1] CRAN (R 3.6.1)
##  DBI           1.1.0     2019-12-15 [1] CRAN (R 3.6.1)
##  dbplyr        1.4.3     2020-04-19 [1] CRAN (R 3.6.3)
##  digest        0.6.25    2020-02-23 [1] CRAN (R 3.6.3)
##  dplyr       * 0.8.5     2020-03-07 [1] CRAN (R 3.6.3)
##  ellipsis      0.3.0     2019-09-20 [1] CRAN (R 3.6.1)
##  evaluate      0.14      2019-05-28 [1] CRAN (R 3.6.1)
##  fansi         0.4.1     2020-01-08 [1] CRAN (R 3.6.2)
##  farver        2.0.3     2020-01-16 [1] CRAN (R 3.6.2)
##  forcats     * 0.5.0     2020-03-01 [1] CRAN (R 3.6.3)
##  fs            1.4.1     2020-04-04 [1] CRAN (R 3.6.3)
##  gdata         2.18.0    2017-06-06 [1] CRAN (R 3.6.1)
##  generics      0.0.2     2018-11-29 [1] CRAN (R 3.6.1)
##  ggplot2     * 3.3.0     2020-03-05 [1] CRAN (R 3.6.3)
##  glue          1.4.0     2020-04-03 [1] CRAN (R 3.6.3)
##  GPArotation   2014.11-1 2014-11-25 [1] CRAN (R 3.6.1)
##  gtable      * 0.3.0     2019-03-25 [1] CRAN (R 3.6.1)
##  gtools        3.8.2     2020-03-31 [1] CRAN (R 3.6.3)
##  haven         2.2.0     2019-11-08 [1] CRAN (R 3.6.1)
##  highr         0.8       2019-03-20 [1] CRAN (R 3.6.1)
##  hms           0.5.3     2020-01-08 [1] CRAN (R 3.6.2)
##  htmltools     0.4.0     2019-10-04 [1] CRAN (R 3.6.1)
##  httr          1.4.1     2019-08-05 [1] CRAN (R 3.6.1)
##  jsonlite      1.6.1     2020-02-02 [1] CRAN (R 3.6.2)
##  knitr       * 1.28      2020-02-06 [1] CRAN (R 3.6.2)
##  labeling      0.3       2014-08-23 [1] CRAN (R 3.6.1)
##  lattice       0.20-38   2018-11-04 [2] CRAN (R 3.6.3)
##  lifecycle     0.2.0     2020-03-06 [1] CRAN (R 3.6.3)
##  lubridate     1.7.8     2020-04-06 [1] CRAN (R 3.6.3)
##  magrittr      1.5       2014-11-22 [1] CRAN (R 3.6.1)
##  mnormt        1.5-6     2020-02-03 [1] CRAN (R 3.6.2)
##  modelr        0.1.6     2020-02-22 [1] CRAN (R 3.6.3)
##  munsell       0.5.0     2018-06-12 [1] CRAN (R 3.6.1)
##  nlme          3.1-147   2020-04-13 [1] CRAN (R 3.6.3)
##  pillar        1.4.3     2019-12-20 [1] CRAN (R 3.6.2)
##  pkgconfig     2.0.3     2019-09-22 [1] CRAN (R 3.6.1)
##  psych         1.9.12.31 2020-01-08 [1] CRAN (R 3.6.2)
##  purrr       * 0.3.4     2020-04-17 [1] CRAN (R 3.6.3)
##  qmethod     * 1.5.5     2020-02-13 [1] CRAN (R 3.6.3)
##  R6            2.4.1     2019-11-12 [1] CRAN (R 3.6.1)
##  Rcpp          1.0.4.6   2020-04-09 [1] CRAN (R 3.6.3)
##  readr       * 1.3.1     2018-12-21 [1] CRAN (R 3.6.1)
##  readxl        1.3.1     2019-03-13 [1] CRAN (R 3.6.1)
##  reprex        0.3.0     2019-05-16 [1] CRAN (R 3.6.1)
##  rlang         0.4.5     2020-03-01 [1] CRAN (R 3.6.3)
##  rmarkdown     2.1       2020-01-20 [1] CRAN (R 3.6.2)
##  rmdformats    0.3.7     2020-03-11 [1] CRAN (R 3.6.3)
##  rstudioapi    0.11      2020-02-07 [1] CRAN (R 3.6.2)
##  rvest         0.3.5     2019-11-08 [1] CRAN (R 3.6.1)
##  scales        1.1.0     2019-11-18 [1] CRAN (R 3.6.1)
##  sessioninfo   1.1.1     2018-11-05 [1] CRAN (R 3.6.1)
##  stringi       1.4.6     2020-02-17 [1] CRAN (R 3.6.3)
##  stringr     * 1.4.0     2019-02-10 [1] CRAN (R 3.6.1)
##  tibble      * 3.0.0     2020-03-30 [1] CRAN (R 3.6.3)
##  tidyr       * 1.0.2     2020-01-24 [1] CRAN (R 3.6.2)
##  tidyselect    1.0.0     2020-01-27 [1] CRAN (R 3.6.2)
##  tidyverse   * 1.3.0     2019-11-21 [1] CRAN (R 3.6.1)
##  utf8          1.1.4     2018-05-24 [1] CRAN (R 3.6.1)
##  vctrs         0.2.4     2020-03-10 [1] CRAN (R 3.6.3)
##  withr         2.1.2     2018-03-15 [1] CRAN (R 3.6.1)
##  xfun          0.13      2020-04-13 [1] CRAN (R 3.6.3)
##  xml2          1.3.1     2020-04-09 [1] CRAN (R 3.6.3)
##  xtable        1.8-4     2019-04-21 [1] CRAN (R 3.6.1)
##  yaml          2.2.1     2020-02-01 [1] CRAN (R 3.6.2)
## 
## [1] /home/ms/R/x86_64-pc-linux-gnu-library/3.6
## [2] /usr/lib/R/library
```
